# Non-native entanglement protein misfolding observed in all-atom simulations and supported by experimental structural ensembles

**DOI:** 10.1126/sciadv.adt8974

**Published:** 2025-08-08

**Authors:** Quyen V. Vu, Ian Sitarik, Yang Jiang, Yingzi Xia, Piyoosh Sharma, Divya Yadav, Hyebin Song, Mai Suan Li, Stephen D. Fried, Edward P. O’Brien

**Affiliations:** ^1^ Institute of Physics, Polish Academy of Sciences, Al. Lotnikow 32/46, 02-668 Warsaw, Poland.; ^2^ Department of Chemistry, Pennsylvania State University, University Park, PA 16802, USA.; ^3^ Department of Chemistry, Johns Hopkins University, Baltimore, MD 21218, USA.; ^4^ Bioinformatics and Genomics Graduate Program, The Huck Institutes of the Life Sciences, Pennsylvania State University, University Park, PA 16802, USA.; ^5^ Department of Statistics, Pennsylvania State University, University Park, PA 16802, USA.; ^6^ Institute for Computational Sciences and Technology, Quang Trung Software City, Tan Chanh Hiep Ward, District 12, Ho Chi Minh City, Vietnam.; ^7^ T. C. Jenkins Department of Biophysics, Johns Hopkins University, Baltimore, MD 21218, USA.; ^8^ Institute for Computational and Data Sciences, Pennsylvania State University, University Park, PA 16802, USA.

## Abstract

Several mechanisms are known to cause monomeric protein misfolding. Coarse-grained simulations have predicted an additional mechanism exists involving off-pathway, noncovalent lasso entanglements, which are long-lived kinetic traps and structurally resemble the native state. Here, we examine whether such misfolded states occur in long-timescale, all-atom folding simulations of ubiquitin and λ-repressor. We find that these entangled misfolded states are populated in higher-resolution models. However, because of the small size of ubiquitin and λ-repressor, these states are short-lived. In contrast, coarse-grained simulations of a larger protein, IspE, predict that it populates long-lived misfolded states. Using an Arrhenius extrapolation applied to all-atom simulations, we estimate that these IspE misfolded states have lifetimes similar to the native state while remaining soluble. We further show that these misfolded states are consistent with the structural changes inferred from limited proteolysis and cross-linking mass spectrometry experiments. Our results indicate that misfolded states composed of non-native entanglements can persist for long timescales in both all-atom simulations and experiments.

## Teaser

All-atom simulations populate a predicted class of misfolding, and ensembles are proposed based on structural mass spectrometry.

## INTRODUCTION

**Fig. 1.**
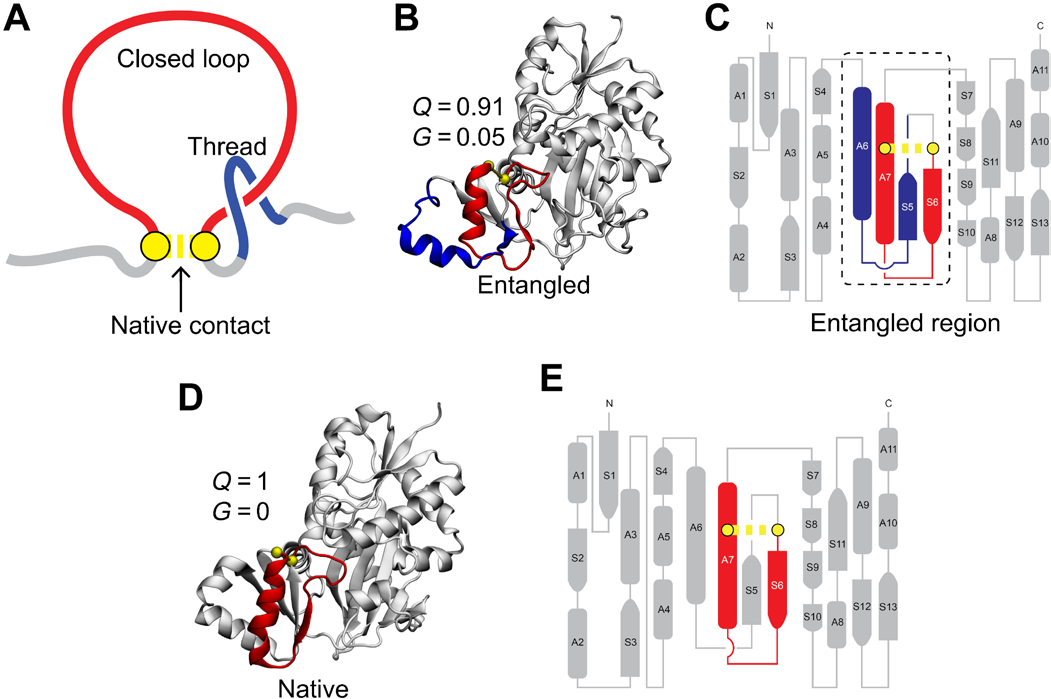
Visualizing changes of entanglement through various representations. (**A**) Illustration of the two geometric elements that compose an entanglement: The closed loop is colored in red, and the threading segment is in blue. The loop is closed by a noncovalent contact between two residues (yellow). Such entanglements occur naturally in the native states of some proteins. In other cases, non-native formation of these entanglements can occur. (**B**) Three-dimensional (3D) structure of a misfolded entangled state from the protein d-alanine—d-alanine ligase B (DDLB; the closed loop and crossing section of the threading segment of their entangled regions are colored in red and blue, respectively) taken from previously reported coarse-grained simulations (*1*). Q and G correspond respectively to the fraction of native contacts formed in the structure and the fraction of native contacts that exhibit a change in entanglement. (**C**) Flattened two-dimensional (2D) structure representation of the entangled state of DDLB. S, β sheet; A, α helix. (**D**) 3D structure of the crystal structure of DDLB. All native contacts are formed in the crystal structure ( Q = 1), and there is no entanglement change (hence, G=0 ). (**E**) Flattened 2D structure representation of the native state of DDLB. The coloring scheme of the relevant elements is the same in all panels.

Various factors are known to cause misfolding in monomeric proteins (Table 1). Recently, high-throughput, coarse-grained simulations of protein synthesis and folding of the *Escherichia coli* proteome have suggested that there exists an additional widespread mechanism of misfolding (*1*–*3*): Proteins can populate off-pathway misfolded states that involve a change in noncovalent lasso entanglements (*1*, *2*). This type of entanglement is defined by the presence of two structural components: a loop formed by a protein backbone segment closed by a noncovalent native contact and another segment of the protein that is threaded through this loop (*4*–*6*) and, in some cases, wrapped around it multiple times (Fig. 1, A and B). Between 49 and 71% of native structures contain a noncovalent lasso entanglement depending on the organism (*7*).

**Table 1.** Mechanisms of protein misfolding. Protein misfolding can result from factors inherent to the amino acid sequence or from external conditions or processes, see references (*56*–*61*).

Intrinsic to the primary structure	Extrinsic to the primary structure
Proline isomerization	Transcription & translation errors
Intrachain domain swapping	mRNA splicing errors
Out-of-register β strands	Perturbation of temperature, oxidative stress, pH, osmotic pressure, protein concentration, chaperones, processing enzymes
Incorrect helix packing	Soluble & insoluble aggregation
Mispacking of side chains	Genetic mutations
Alternative secondary or tertiary structure	Erroneous complex formation
Backtracking (frustration)	Improper/incomplete degradation
Incorrect disulfide bond formation	Cofactor and ligand concentrations
	Posttranslational modifications
	Interchain domain swapping

The misfolded states observed in the coarse-grained simulations involved either the gain of a non-native entanglement (i.e., the formation of an entanglement that is not present in the native ensemble; table S1 and fig. S1) or the loss of a native entanglement (i.e., an entanglement present in the native state fails to form; table S1 and fig. S1) (*1*–*3*). This predicted class of misfolding offers an explanation for the decades-old observations that nonfunctional (or less functional) protein molecules can persist for long timescales without aggregating and, in the presence of chaperones, neither get refolded nor degraded (*8*–*10*).

The coarse-grained simulations, in which individual residues are represented by single interaction sites, suggest that these misfolded states are often long-lived because, to correctly fold, they need to change their entanglement state: For example, an entangled state would need to disentangle, which is energetically costly as correctly folded portions of the protein would need to unfold (see folded gray segments in Fig. 1, B and C). Moreover, these states are more likely to bypass cellular quality control mechanisms and remain long-lived in vivo (*8*–*10*) because, in some cases, they are similar in size and structure to the native state and do not expose that much more hydrophobic surface (compare Fig. 1, B and D).

Two criticisms of these findings are that they are based on a model with limited spatial resolution and use force field approximations that have the potential to affect the results. It could be the case, for example, that these states are never populated in a more detailed, transferable all-atom model. Furthermore, the coarse-grained force field previously used was “structure-based,” meaning that the native state is encoded to be favored over other states (*11*). Both approximations have the potential to affect the accuracy of the earlier findings.

Here, we examine whether an all-atom model of the protein folding process—based on a transferable force field—also exhibits these self-entangled states, as observed previously in coarse-grained models; and if they do, we test whether those states are off-pathway long-lived traps with properties similar to the native state. We show that this type of misfolding occurs in all-atom simulations and that for typically sized proteins, these misfolded states can be very long-lived, soluble kinetically trapped states and then combine information from mass spectrometry and simulations to propose experimentally informed misfolded ensembles.

## RESULTS

### Ubiquitin and λ-repressor exhibit short-lived entangled states

**Fig. 2.**
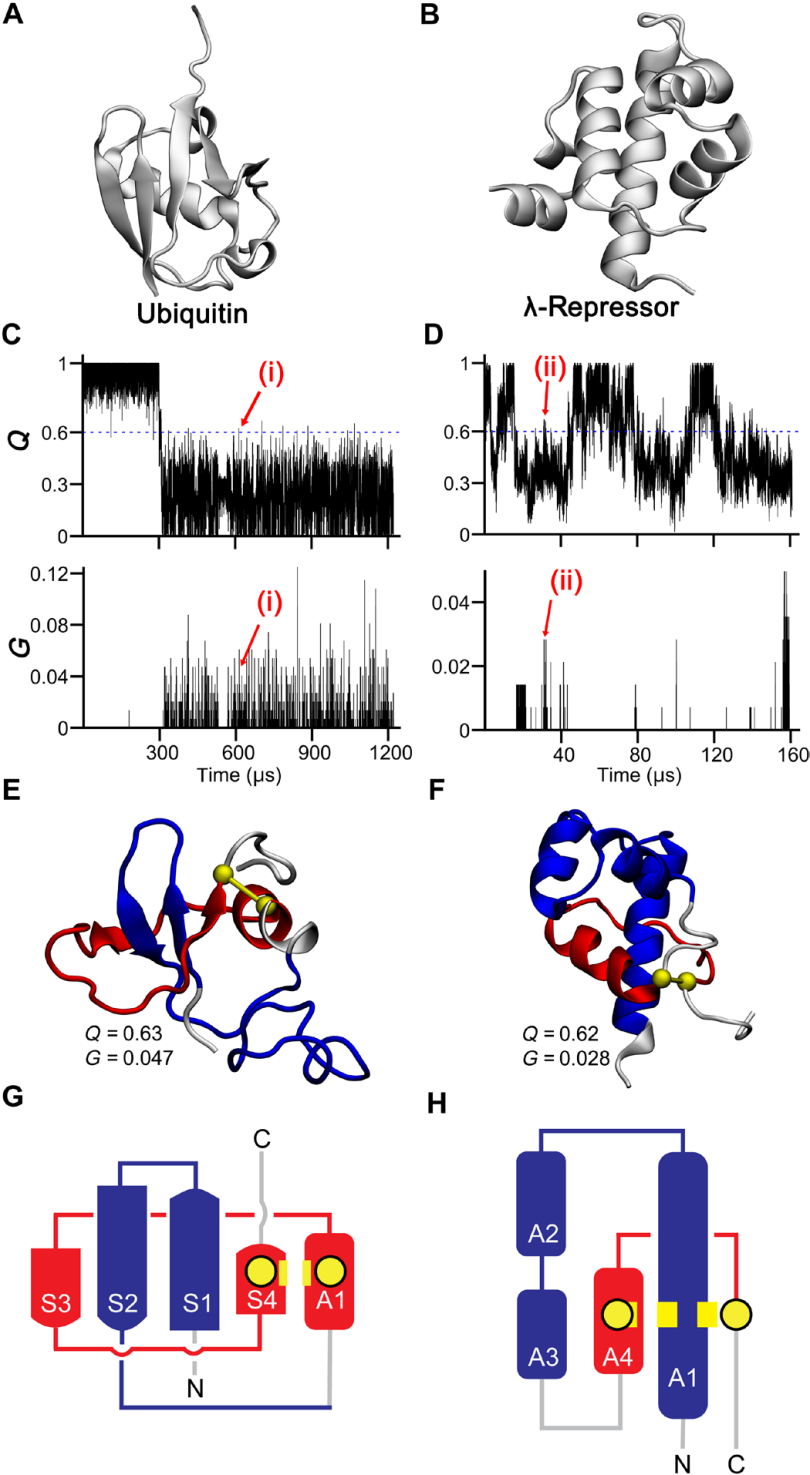
Misfolded gains of entanglement are observed in all-atom protein folding simulations. (**A**) Ubiquitin’s native structure and (**B**) λ-repressor’s native structure have no entanglement present. (**C**) Upper panel: ubiquitin’s fraction of native contacts Q versus time from all-atom simulations. The blue dashed line represents Q=0.6 . Lower panel: ubiquitin’s degree of entanglement G versus time for the same trajectory. (**D**) Same as (C) but for λ-repressor. (**E**) 3D structure of a ubiquitin entangled state observed at the time point labeled (i) in (C), with entanglement elements colored as in Fig. 1A. (**F**) Same as (E) but for λ-repressor and from point labeled (ii) in (D). (**G**) Flattened secondary structure representation for the entangled state in (E). (**H**) Same as (G) but for λ-repressor.

To address these questions, we analyze previously reported all-atom protein folding trajectories of ubiquitin (*12*) and the N-terminal domain (NTD) of λ-repressor (*13*) starting from their native and unfolded states (Fig. 2, A and B). Ubiquitin is a small protein (76 residues) found in eukaryotic organisms and regulates a range of processes including protein degradation and the cell cycle. Ubiquitin folds on the millisecond timescale under typical conditions in vitro (*14*) and in 3 ms in molecular dynamics simulations (*12*). The NTD of λ-repressor in the published trajectories (*13*) is 80 residues in length, binds DNA, and folds on the microsecond timescale (*13*, *15*). Noncovalent lasso entanglements are not present in the native structure of either of these proteins.

We analyzed whether non-native entanglements are populated during the folding trajectories of ubiquitin and λ-repressor totaling 8 ms and 643 μs of simulation time, respectively. To characterize the folding events in the simulations, we used the fraction of native contacts ( Q ) and the order parameter G (defined in Eq. 4, which measures the fraction of native contacts that exhibit a change in entanglement). We observed several non-native entangled states (denoted by nonzero G values, shown in Fig. 2, C and D) during folding transitions, which are indicated by shifts from low to high Q values. Thus, protein self-entangled states are observed in all-atom models. Next, we asked whether these entangled states are off-pathway. We never observe in these trajectories a direct transition from entangled clusters (clusters 1 and 4 for ubiquitin and clusters 2 and 4 for λ-repressor from structural clustering; fig. S2) to the native cluster (clusters 6 and 5, respectively, for ubiquitin and λ-repressor). Hence, these misfolded states are off-pathway—they must unfold to reach the native state (as indicated in fig. S2, these entangled structures always transition to clusters with lower Q and G ). These entangled states clearly are short-lived, lasting just a few nanoseconds according to Fig. 2 (C and D). This is seemingly at odds with the coarse-grained model prediction that these can be long-lived states (*2*). However, these all-atom simulations were performed at high temperatures (390 K for ubiquitin and 350 K for λ-repressor), near their melting temperatures in the all-atom force field, and configurational transitions are accelerated. Therefore, these entangled states may be able to rapidly disentangle because of these high temperatures.

To test whether these entangled states are near-native, kinetic traps at physiological temperatures, we performed unrestrained molecular dynamics simulations at 310 K (37°C). We selected entangled structures that had at least 60% of their native contacts formed as starting structures, resulting in 21 and 12 structures, respectively, for ubiquitin and λ-repressor. Structurally, these entangled structures are qualitatively similar to each other (table S2); 20 of the 21 ubiquitin structures have closed loops located toward the C terminus, and the threading segment is composed of the N-terminal portion. For λ-repressor, the loop forms toward the C-terminal end and the N terminus threads through it in all 12 structures. Representative structures for these entangled states are shown in Fig. 2 (E and F). Three independent trajectories were started from each of these conformations, and the simulations were run for 700 ns or until the entanglement was lost (i.e., a value of G=0 was reached).

For the 63 trajectories (= 21 × 3) of ubiquitin, 71% (45 of 63) of trajectories persist for 700 ns (an example in fig. S3A and detailed in table S2). On average then, the time to disentangle is ~2.1 μs (estimated using Eq. 6, setting t=700 ns). For λ-repressor, 17% of trajectories (6 of 36) persist in an entangled state for 700 ns (an example is shown in fig. S3B and table S2). The average time to disentangle for λ-repressor is ~390 ns, 50 times faster than for ubiquitin entanglements.

The radii of gyration of these entangled states are comparable to the native state (the differences are less than 10%; table S3), indicating that the entangled and native states are of similar sizes. These entangled states are estimated to have solubility similar to the native state (table S3) according to a model that accounts for the chemical properties of the exposed protein surface (*1*). Next, we analyzed the secondary structure of proteins using Stride software (*16*) and found that the non-native entangled states contain up to 83% of the secondary structure found in the native state (table S2). Thus, the size, solubility, and secondary structure analyses indicate that these misfolded states are similar to the native state.

Relative to their protein folding timescales, these entangled states are not long-lived kinetic traps even at 310 K. On the basis of the previous coarse-grained simulation results, this is to be expected (*1*), because ubiquitin and the NTD of λ-repressor are small single-domain proteins. They are representative of the “model proteins” that have been traditionally studied by biophysicists and demonstrate the ability to rapidly and reversibly unfold and refold (*17*) but are not representative of the complexity of proteomes (*18*) [for example, the median protein length among eukaryotes is 361 residues and that among archaea is 247 residues (*19*)]. In such small proteins, entanglements comprise a large proportion of the total protein structure present—and hence, it is easier to disentangle since most of the protein structure is misfolded and less stable. For example, for the entanglements reported in table S2, up to 78% of ubiquitin’s primary structure is involved in the entanglement (i.e., the minimum distance along the primary sequence required to maintain the entanglement; Fig. 2, G and H), and up to 84% of λ-repressor’s primary structure is involved in the entanglement. In contrast, when a protein of median length misfolds, a larger proportion of the already folded structure will need to unfold to permit disentanglement (Fig. 1B). Unfolding the correctly folded portions of these proteins is energetically more costly, and hence, misfolded states involving non-native entanglements in typically sized proteins are more likely to be long-lived kinetic traps (*2*).

### Misfolded states in a typically sized protein, IspE, are long-lived states in all-atom simulations

**Fig. 3.**
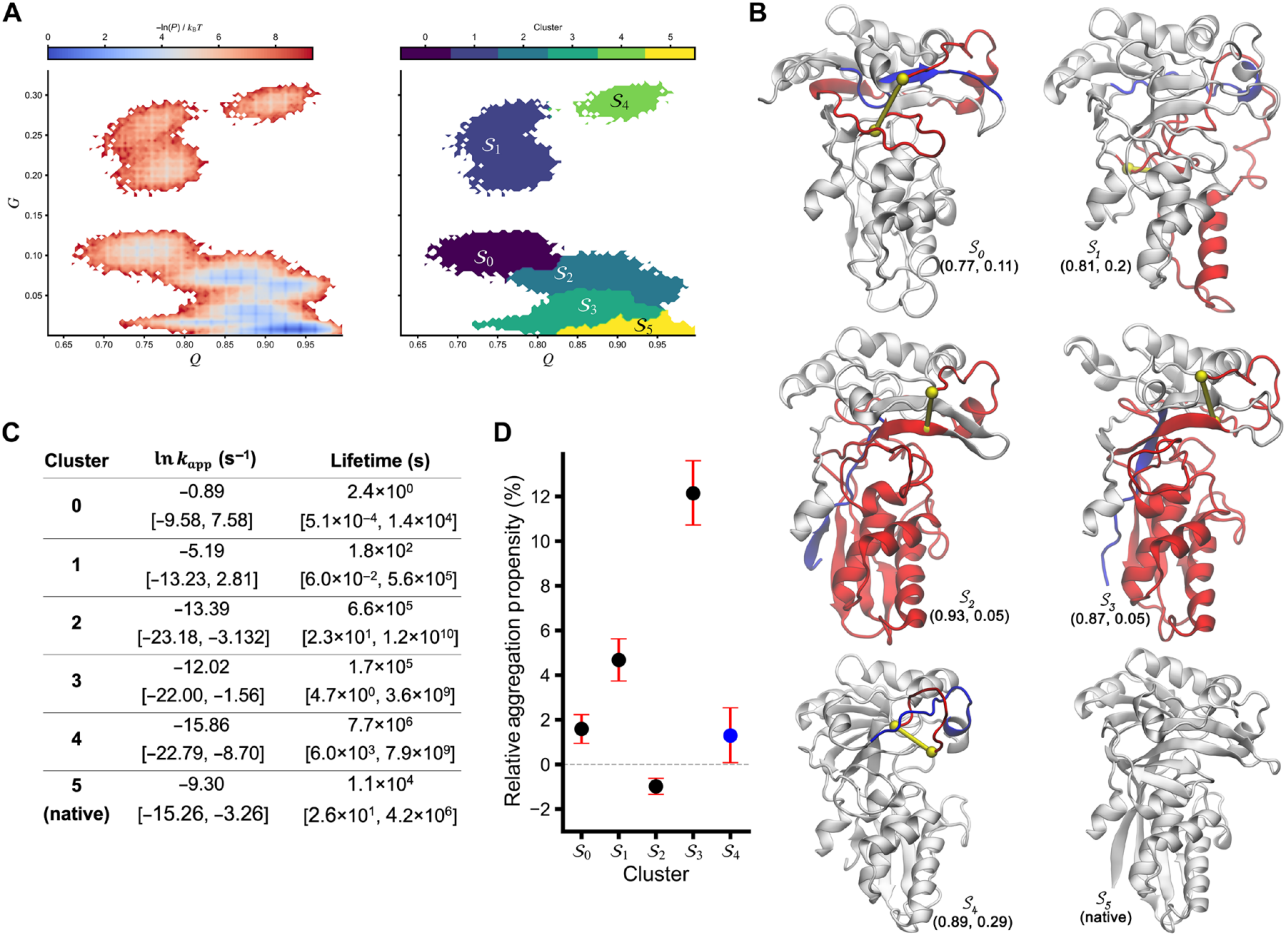
IspE misfolded states persist for long periods while remaining soluble. (**A**) −ln(*P*) surface and structural clusters identified from clustering analysis. (**B**) Structures selected from each cluster for all-atom temperature jump simulations. *Q* and *G* values of the structure are reported below the structure. Entanglement elements colored as in Fig. 1A. Interactive visualizations of these structures are available at https://obrien-lab.github.io/All_Atom_Misfolding_Exp_Ensemble/. Additional structures from each cluster can be found in data S2. (**C**) Apparent unfolding rates and estimated lifetimes of the structures in (B), derived from Arrhenius extrapolation. (**D**) Relative aggregation propensities of misfolded clusters compared to the native state. The aggregation propensity of cluster 4 is not significantly different from that of the native cluster, which is indicated by the blue circle. Error bars in (C) and (D) represent the 95% confidence intervals.

Unrestrained all-atom simulations are incapable of folding such large proteins on tractable simulation timescales. Therefore, to test the aforementioned “size-effect” hypothesis, we calculated the lifetime of misfolded states of a larger protein, *E. coli*’s 4-diphosphocytidyl-2-*C*-methyl-d-erythritol kinase (283 residues; gene *ispE*), within an aqueous, all-atom simulation model. This protein was chosen because it was previously identified to exhibit entangled misfolded states in coarse-grained simulations (*2*, *11*). To create models of misfolded IspE for all-atom simulations, we (i) performed protein folding simulations in a coarse-grained model; (ii) carried out structural clustering; (iii) scored the structures within each cluster for their consistency with limited proteolysis mass spectrometry (LiP-MS) and cross-linking mass spectrometry (XL-MS) data (described in the next section and Materials and Methods); (iv) chose, within each misfolded cluster, the single structure that had the highest score; and (v) backmapped those structures to an all-atom representation. These backmapped structures were then used as starting conformations for the aqueous, all-atom temperature jump simulations that were used to estimate their lifetimes at 298 K using an Arrhenius analysis. Five distinct misfolded clusters are observed in the simulations of this enzyme (step ii; Fig. 3A), and their backmapped all-atom representative structures (step v) are visualized in Fig. 3B. An Arrhenius analysis [i.e., performing unfolding simulations at high temperatures (600, 650, 700, 750, and 800 K) and extrapolating the unfolding time to 298 K] is necessary because all-atom simulations cannot reach the timescales associated with unfolding at low temperatures. The resulting unfolding rates exhibited super-Arrhenius behavior (figs. S5 and S6) (*20*) and were fit to Eq. 11 to estimate the unfolding rate at 298 K. The lifetimes were obtained as the inverse of the extrapolated unfolding rates.

To determine whether these misfolded structures are long-lived, we compared their estimated lifetimes to the lifetime of the native cluster in the simulation model. (There are no experimentally reported lifetimes of native IspE; hence, we had to use the lifetime of native IspE within the model.) We find that the native cluster is estimated to have a lifetime of 3 hours (Fig. 3C), corresponding to an unfolding rate of 9.1 × 10^−5^ s^−1^. In contrast, structures from clusters 2 to 4 persist for days to months (Fig. 3, B and C), while less structured clusters 0 and 1 last only seconds to minutes. We emphasize that because of the large uncertainties in these lifetime estimates (see 95% confidence intervals in Fig. 3C), only qualitative conclusions can be drawn that are relative to the estimated lifetime of the native cluster. We conclude that in all-atom simulations, misfolded clusters 2 to 4 are likely to be kinetic traps with lifetimes of a similar order of magnitude to the native cluster.

### IspE misfolds for over an hour in refolding experiments

We next experimentally tested whether IspE populates misfolded states. To address this, we performed LiP-MS (*21*–*24*) and XL-MS (*25*–*27*) on IspE after denaturing and refolding, and compared it to a native IspE sample that was never denatured to detect structural changes such as those associated with changes in entanglement (*2*, *28*). Refolded samples were prepared by first chemically denaturing IspE and then refolding it by dilution from high to low denaturant concentration. Then, the sample was incubated at room temperature for 1 hour before being exposed either to proteinase K (PK) in the LiP-MS experiment or to the cross-linking agent in the XL-MS experiment. We then detect differential signal changes between native (untreated) samples and refolded (treated) samples. We define a signal as significantly different if it exhibits more than a twofold difference in the mean abundance between the refolded and native samples ( $|\;{\text{log}}_{2}\text{FC}\;\left(\text{fold change}\right)\;|\geq 1$ ) and an adjusted *P* value, false discovery rate corrected—applied separately to the XL-MS and LiP-MS datasets—using the Benjamini-Hochberg method (*29*), of less than or equal to 0.05. We observe that there are changes in the proteolytic susceptibility and cross-linking patterns between chemically renatured IspE 1 hour after refolding conditions have been established and a native reference that was not denatured. Specifically, we identified 13 PK cleavage sites from LiP-MS (Table 2 and data S1, sheet labeled “EXP-LiPMS”) and 20 cross-links from XL-MS (Table 3 and data S1, sheet labeled “EXP-XLMS”) experiments that exhibited significant differences in abundance between refolded and native samples. These results suggest that in the case of LiP-MS, the solvent exposure of these regions along the primary structure of IspE was significantly altered in the refolded sample, and in the case of XL-MS, structural changes in the protein led to altered cross-linking propensity between various pairs of residues. We therefore conclude that IspE can populate soluble misfolded conformations over long timescales, qualitatively consistent with our simulation predictions.

**Table 2.** PK cut sites exhibiting significant differences in abundance between refolded and native samples identified by LiP-MS.

PK site	$|{\text{log}}_{2}\text{FC}|$ ^*^	Adj.P value ^†^	PK site	$|{\text{log}}_{2}\text{FC}|$ ^*^	Adj.P value ^†^
**R69**	1.78	0.028	**F207**	2.60	0.000
**M75**	4.14	0.007	**A254**	2.15	0.016
**A78**	2.17	0.000	**R255**	1.53	0.033
**I159**	2.14	0.002	**L258**	1.90	0.009
**L202**	1.46	0.007	**Q260**	2.47	0.018
**K204**	1.74	0.038	**A261**	2.67	0.004
**E206**	1.82	0.003			

*FC: fold change defined as the ratio of the mean refold-sample abundance to native-sample abundance across five replicates each. †Adj. *P* value: Benjamini-Hochberg adjusted *P* value.

**Table 3.** Cross-links exhibiting significant differences in abundance between refolded and native samples identified by XL-MS.

Cross-link	$|{\text{log}}_{2}\text{FC}|$ ^*^	Adj.P value ^†^	Cross-link	$|{\text{log}}_{2}\text{FC}|$ ^*^	Adj.P value ^†^
**S7-Y16**	1.54	0.050	**K96-K196**	5.33	0.000
**K10-K196**	7.24	0.003	**K96-S208**	5.33	0.001
**Y16-K196**	2.72	0.003	**T161-K204**	2.19	0.042
**K76-T86**	4.53	0.000	**K196-S198**	1.72	0.000
**K76-T161**	11.93	0.008	**K196-K204**	3.90	0.000
**K76-K196**	1.85	0.000	**K196-S208**	6.83	0.000
**K76-S208**	5.55	0.014	**K196-K271**	2.93	0.000
**T77-S208**	4.74	0.016	**K196-S276**	1.34	0.042
**S81-T86**	1.20	0.002	**S198-S208**	3.87	0.024
**S81-K96**	1.88	0.000	**T201-S208**	1.60	0.001

*FC: fold change defined as the ratio of the mean refold-sample abundance to native-sample abundance across five replicates each. † Adj. *P* value: Benjamini-Hochberg adjusted *P* value.

### Misfolded structural ensembles of IspE that are consistent with LiP-MS and XL-MS

With 33 unique experimental signals reporting on changes in structure in IspE relative to the native state, we can examine which misfolded states seen in our simulations are consistent with these signals. To do this, we take a two-stage approach: First, we simulated the refolding of IspE in our coarse-grained model, and second, we scored the resulting misfolded subpopulations for their overall consistency with the mass spectrometric data (see Materials and Methods). From the simulations, we structurally clustered the resulting conformations and found one cluster corresponding to the native ensemble and five distinct misfolded clusters (Fig. 3A) that vary in their degree of native-structure formation (*Q*) and changes in entanglement status (*G*). Scoring each misfolded cluster in terms of its relative change in solvent accessibility for LiP-MS results and solvent-accessible surface distance (SASD) [as calculated with JWalk (*30*)] for XL-MS results, we find that cluster 3 is the highest scoring with 22 of 33 experimental signals consistent with the structural changes observed in this cluster. (For LiP-MS and XL-MS, respectively, 7 of 13 and 15 of 20 are consistent; data S1 labeled “Comparison-LiPMS” and “Comparison-XLMS”). In comparison, cluster 0 is consistent with 14 of the signals, cluster 2 with 11, and cluster 1 with 9. Cluster 4, which is the most similar to the native cluster, is consistent with only four of the signals.

Across the five misfolded clusters, 29 unique experimental signals can be explained by the structural changes observed in this combination of clusters. One LiP-MS and three XL-MS signals cannot be explained by our methodology. Thus, 88% of the experimental signals can be explained by this ensemble of five misfolded clusters. For interactive visualization, we provide structures from each misfolded cluster in PDB format in data S2.

### Most IspE misfolded states are likely to stay soluble

Although most of these misfolded states are long-lived, we next examine whether they are likely to remain soluble or aggregate. To address this, we estimated the relative aggregation propensity of these misfolded clusters compared to the native cluster by incorporating information on the solvent accessibility of aggregation-prone regions (Eq. 14). We first used the AMYLPRED2 server (*31*) to identify regions in IspE that are prone to aggregation when exposed to solvent. We then calculated the solvent-accessible surface area (SASA) of these regions from our simulated trajectories for each misfolded and native cluster. Our analysis indicates that the aggregation propensity of these misfolded clusters is very similar to that of the native cluster (Fig. 3D), with differences of less than 5%—except for cluster 3, which exhibits a 12.15% relative increase to the native state. Although these small differences are statistically significant (Benjamini-Hochberg adjusted *P* value ≤ 0.05) for all clusters except cluster 4, the small effect sizes (<5% increase) suggest that most of these misfolded ensembles would remain soluble on timescales similar to the native ensemble.

## DISCUSSION

Coarse-grained simulations predicted the existence of an additional widespread mechanism of protein misfolding. Motivated by the possibility this may be an artifact of those simulations’ resolution and structure-based force field, in this study, we examined whether protein folding modeled at all-atom resolution also exhibits this type of misfolding. We have demonstrated that ubiquitin and λ-repressor populate these entangled misfolded states in all-atom folding simulations. Thus, protein folding involving a change of entanglement status occurs regardless of model resolution, lending support to the validity of the earlier conclusions drawn from coarse-grained modeling.

Because of the small size of ubiquitin and λ-repressor, the entangled states they populate are short-lived, persisting for just a few microseconds. For larger proteins, all-atom simulations indicate that these misfolded states can persist for much longer periods—on timescales similar to the lifetime of the native state while remaining soluble. This relationship between protein size and misfolded lifetimes arises from the fact that a more stabilizing native structure can form around the localized misfolded elements in larger proteins as compared to smaller proteins, creating a larger energy barrier to unfold improperly folded portions.

Experimentally, we observed that IspE can populate misfolded structures that form after dilution from the denaturant and remain misfolded for over an hour. This result is consistent with our simulations of IspE (Fig. 3, A to C), giving us more confidence in our broader conclusions. When cross-referencing the misfolded signals from LiP-MS and XL-MS experiments with the misfolded clusters from the IspE coarse-grained simulations, we find that almost 90% of the experimental signals can be explained by the simulated structural changes. Thus, the structures we have identified in Fig. 3B and data S2 represent an experimentally informed misfolded ensemble involving soluble, long-lived states.

Misfolding involving changes of entanglement status can arise from either a gain of a non-native entanglement or a loss of a native entanglement. In ubiquitin and λ-repressor, which contain no noncovalent lasso entanglements in their native state, it is only possible to misfold through the formation of a non-native entanglement (gain of entanglement), which we observed during their folding. In contrast, IspE has two unique representative, noncovalent lasso entanglements present in its native structure (table S5). In this system, we observed misfolded structures involving either gains of entanglement, losses of entanglement, or combinations thereof. Both classes of misfolding are manifestations of topological frustration. In the one case, a protein backbone segment is improperly positioned outside a backbone loop, corresponding to a loss of entanglement; in the other case, it is improperly positioned within a backbone loop, corresponding to a gain of entanglement. The consequence of both of these types of topological traps is that they both must backtrack (*32*) out of these states, meaning that already natively structured portions of the protein must unfold to allow proper folding. Small proteins that misfold in this way have additional folding pathways that bypass backtracking that include threading of the loop in cases in which the native entanglement failed to form or unthreading of a loop in misfolded states involving formation of non-native entanglements (*1*, *33*). However, for typically sized proteins, such as IspE, backtracking will often be the dominant pathway because substantial portions of the structure can still properly fold.

These changes of entanglement that lead to misfolded, long-lived kinetic traps expand the mechanisms of topological frustration that give rise to the kinetic partitioning mechanism for understanding biomolecular self-assembly in proteins and nucleic acids (*34*–*36*). In the kinetic partitioning model, frustration is inherent to the nature of biopolymers, because interactions at local and global length scales can be in energetic conflict, giving rise to different structural preferences. Particular packing arrangements, for example, of hydrophobic residues or secondary structural elements might be energetically favored locally but disfavored in the presence of tertiary interactions (*34*). As a consequence, near-native misfolded states can be populated, especially for larger proteins, which will tend to exhibit greater frustration. The current study adds to the structural mechanisms by which such frustration can arise, specifically through the formation of native-like states that either fail to form their native entanglement or form a non-native entanglement.

Proteins containing entanglements in their native state are widespread across different organisms. The proportion of globular proteins with them ranges from 49% in humans to 71% in *E. coli* (*7*). Perhaps expectedly then, this type of misfolding has been found to be common based on high-throughput coarse-grained simulations of the *E. coli* proteome (*2*). Furthermore, because structure equals function, such misfolding has been implicated in influencing ensemble-averaged functioning of proteins like specific activity (*1*) and, in some cases, in bypassing the chaperone machinery in cells depending on how native-like in structure are these misfolded states (*3*). We hypothesize that this understudied class of protein misfolding will offer insights into protein function and homeostasis, including new causal mechanisms for disease and aging that can open up new therapeutic targets.

## MATERIALS AND METHODS

### All-atom simulations of ubiquitin and λ-repressor

Initial entangled conformations of the protein were placed at the center of a rectangular periodic box with the minimum distance between the box edge and protein atoms of 1.5 nm, the system was solvated with the TIP3P water model (*37*), and NaCl was added to neutralize and mimic relevant salt concentrations [ubiquitin: 0 mM; λ-repressor: 50 mM; the salt concentration was added according to original studies (*12*, *13*)]. Energy minimization was then carried out using the steepest descent algorithm (*38*, *39*) to minimize steric conflict. The systems were then gradually heated from 2 to 310 K over 1 ns. After that, the system was equilibrated for 1 ns in the NVT ensemble and 1 ns in the NPT ensemble with all heavy atoms restrained using a harmonic potential with a force constant of 1000 kJ/mol per square nanometer before it went into production run in the NPT ensemble. The particle mesh Ewald method (*40*) was used to calculate long-range electrostatic interactions beyond 12 Å, and the Lennard-Jones interactions were calculated with a cutoff distance of 12 Å and applied, smoothly switching the forces to zero between 10 and 12 Å. Temperature and pressure were maintained at 310 K and 1 atm using a Nose-Hoover thermostat (*41*, *42*) and Parrinello-Rahman barostat (*43*), respectively. The LINCS algorithm (*44*) was used to constrain all bonds involving hydrogen atoms, and the integration time step was set to 2 fs. Simulations were performed using GROMACS 2018 (*45*) with the CHARMM36m force field (*46*). This procedure was used in all the new simulations carried out in this study.

### Characterizing changes in entanglement

Calculating changes in entanglement has been reported elsewhere (*1*–*3*). Here, we briefly describe our procedure. To detect noncovalent lasso entanglements, we use linking numbers, which require at least one closed loop. We define this loop as being composed of the backbone trace connecting residues 𝑖 and 𝑗 that form a noncovalent contact in the given protein conformation. The looped portion (red segment in Fig. 1A) is identified if the C_α_ coordinates of residues 𝑖 and 𝑗 (yellow spheres in Fig. 1A) are closer than 8 Å and ∣i−j∣≥10 residues. Outside this loop is an N-terminal segment composed of residues 1 through 𝑖 − 1, and there is a C-terminal segment composed of residues 𝑗 + 1 through 𝑁. These two segments represent open curves, whose entanglement through the closed loop was characterized by linking numbers denoted 𝑔_N_ and 𝑔_C_. For a given structure of an *N*-residue protein, with a contact present at residues (𝑖, 𝑗), the average coordinates **R**_𝑙_ and the gradient *d***R**_𝑙_ of point 𝑙 on the curves were calculated as

{Rl=12(rl+rl+1)dRl=rl+1−rl (1)

where **r**_𝑙_ is the coordinates of the C_α_ atom in residue 𝑙. The linking numbers 𝑔_N_(𝑖, 𝑗) and 𝑔_C_(𝑖, 𝑗) of N and C termini, respectively, were calculated as

{gN(i,j)=14π∑m=6i−5∑n=ij−1Rm−Rn∣Rm−Rn∣3(dRm×dRn)gC(i,j)=14π∑m=ij−1∑n=j+4N−6Rm−Rn∣Rm−Rn∣3(dRm×dRn) (2)

where we excluded the first five residues on the N-terminal curve, the last five residues on the C-terminal curve, and four residues before and after the native contact to eliminate the error introduced by both the high flexibility and contiguity of the termini and trivial entanglements in the local structure. It is worth noting that partial linking values between 0.5 and 0.7 may not signify real entanglements and, thus, those have to be checked manually (*47*). The above integrations yield two noninteger values, and the total linking number for a contact (𝑖, 𝑗) is estimated as the sum of N- and C-terminal linking numbers

$$g\left(i,j\right)=\text{round}\left[g_{N}\left(i,j\right)\right]+\text{round}\left[g_{C}\left(i,j\right)\right]$$ (3)

The use of the round function in Eq. 3 serves to discretize the computed linking numbers, which can result in noninteger values because of numerical integration between a closed loop and an open segment. By applying the round function, we map such values to discrete integers (for example, *g* = 0.6 → 1), effectively capturing the presence or absence of an entanglement in a robust and consistent way. This discretization allows us to perform meaningful comparisons between the entanglement states of different structures. For instance, even if both a conformation in the simulated trajectory and the native structure are entangled, their computed linking values may slightly differ (for example, *g* = 0.6 versus *g* = 0.7), yet both indicate the same qualitative topological feature (i.e., one event of terminal threading). The round function ensures that such topologies are treated identically in our analysis.

Comparing the absolute value of the total linking number for a contact (𝑖, 𝑗) at a given conformation to that of a reference state (i.e., native state) allows us to ascertain a gain or loss of linking between the backbone trace loop and the terminal open curves as well as any switches in chirality. There are six changes in linking cases we should consider (table S1 and fig. S1) when using this approach to quantify entanglement.

We defined the *G* metric, the degree of entanglement, as a time-dependent order parameter that reflects the extent of the topological entanglement changes in a given structure compared to the native structure and is calculated as

$$G\left(t\right)=\frac{1}{M}\sum_{\left(i,j\right)}\Theta \left[\left(i,j\right)\in \text{NC}⋂g\left(i,j,t\right)\neq g^{\text{native}}\left(i,j\right)\right]$$ (4)

where (𝑖, 𝑗) is one of the native contacts in the native crystal structure; NC is the set of native contacts formed in the current structure at time 𝑡; 𝑔(𝑖, 𝑗, 𝑡) and 𝑔^native^(𝑖, 𝑗) are, respectively, the total linking number of the contact (𝑖, 𝑗) at time 𝑡 and native structures estimated using Eq. 3; *M* is the total number of native contacts within the native structure; and the selection function Θ equals 1 when the condition is true and equals 0 when it is false. The larger *G* is, the greater the number of native contacts that have changed their entanglement status relative to the reference state.

### Disentanglement time constant from unrestrained simulations at physiological temperature

The disentangled time constant can be estimated through the connection with the portion of disentangled trajectories

$$P_{\text{dis}}\left(t\right)=1-e^{\frac{-t}{\tau }}$$ (5)

$$\tau =\frac{-t}{\text{ln}\left[1-P_{\text{dis}}\left(t\right)\right]}$$ (6)

where $P_{\text{dis}}\left(t\right)$ is the portion of disentangled trajectories at time *t*. τ is a time constant that entanglement disappears. We use this equation to estimate the average lifetime of entangled states of ubiquitin and λ-repressor by setting *t* = 700 ns.

### Secondary structure similarity definition

The secondary structure similarity in table S2 is defined as the fraction of residues that are in the correct secondary element at the current structure compared to the native structure; we only count residues in the α helix or β sheet. The secondary structural elements of protein are assigned by the STRIDE program (*16*).

### Calculation of solubility of the entangled structures

The solubility of the entangled structures in table S3 is defined as the percent soluble protein estimated via the insolubility propensities of structure 𝑖 ( $\chi_{i}^{\text{sol}}$ ), a fully disordered structure, which has no secondary and tertiary structure elements ( $\chi_{\text{disordered}}^{\text{insol}}$ ), and the minimum propensity values of all structures [ $\text{min}\left(\chi_{i}^{\text{sol}}\right)$]

$$f_{i}^{\text{sol}}=\frac{\chi_{\text{disordered}}^{\text{insol}}-\chi_{i}^{\text{insol}}}{\chi_{\text{disordered}}^{\text{insol}}-\text{min}\left(\chi_{i}^{\text{insol}}\right)}$$ (7)

The insolubility was estimated by considering the aggregation propensity ( $\chi^{\text{agg}}$ ) and degradation propensity ( $\chi^{\text{deg}}$ ) minus the 70-kDa heat shock protein (Hsp70) binding propensity ( $\chi^{\text{Hsp}70}$ ) that is considered to prevent the misfolding protein from aggregation

$$\chi^{\text{insol}}=\chi^{\text{agg}}+\chi^{\text{deg}}-\chi^{\text{Hsp}70}$$ (8)

For a given entangled structure, the aggregation, degradation, and Hsp70 binding propensities were estimated as the relative change of the SASA of the aggregation-prone, degradation-prone, and Hsp70 binding regions of the entangled state 𝑖 against the SASA of the native structure

$$\chi_{i}^{T}=\frac{{\text{SASA}}_{i}^{T}}{{\text{SASA}}_{\text{native}}^{T}}-1$$ (9)

where T can be aggregation, degradation, or Hsp70 binding propensities. The aggregation-prone region was predicted by the AmylPred2 server (*31*). The degradation-prone region was defined as the hydrophobic residues (Ile, Val, Phe, Cys, Met, Ala, Gly, and Trp). The Hsp70 binding region was predicted by using ChaperISM (*48*).

### Coarse-grained protein folding simulations of IspE protein and structural clustering

We use a previously published Go̅-based coarse-grain model (*1*) in which each amino acid is represented by a single interaction site placed at the C_α_ atom with a specific van der Waals radius for each amino acid. Initial conformations for refolding simulations were generated by heating the native state of the protein to 1000 K for 15 ns. The final conformation from heating was then temperature quenched at 310 K to initialize refolding. All simulations were carried out using a Langevin thermostat at a temperature of 310 K, with a time step of 15 fs and a friction coefficient of 0.050 ps^−1^. All simulations were carried out using OpenMM version 7.7 (*49*). Trajectories were saved after every 5000 steps; a total of 56 independent trajectories of 1.5 μs was performed. The last 500 ns from each trajectory was used for structural clustering on the basis of two order parameters that capture the nativeness of the structures (fraction of native contacts, *Q*) and the changes in self-entanglement of the protein (fraction of native contact change in entanglement, *G*). The fraction of native contacts at each conformation was defined as the number of native contacts formed divided by the total number of native contacts in the crystal structure. Two residues (𝑖, 𝑗) were considered to form a native contact if the distance between their C_α_ atoms was less than 1.2 times their distance in the crystal structure, and ∣i−j∣>3 . The fraction of native contact change in entanglement (*G*) was calculated as described in Eq. 4. Using the K-mean++ clustering algorithm (*50*), 100 microstates were grouped from the coarse-grained simulations. These microstates are then coarse grained into structural states using the PCCA+ algorithm (*51*). To determine a reasonable number of clusters for the PCCA+ analysis, we used a timescale separation criterion based on identifying a large gap in the implied timescale (ITS) spectrum derived from eigenvalues of the transition probability matrix (*52*). Specifically, we calculated the ITSs, sorted them in descending order, and computed the ITS ratio, defined by the ratio ${\text{ITS}}_{i}/{\text{ITS}}_{i+1}$ . A large ratio indicates that the corresponding dynamical processes are kinetically well separated, while a ratio close to 1 suggests that the processes can be grouped together. All clustering analyses were performed by using the PyEmma package (*53*).

### Structure selection for Arrhenius analyses

The last 500-ns simulation structures were labeled using the cluster ID and the experimental signals they produce. Here, we identify a simulated structure capable of generating an experimental signal only if it demonstrates a SASA greater than the 97.5th percentile or less than the 2.5th percentile of all structures in the native ensemble for a LiP-MS signal or exhibits a cross-linking propensity score outside the corresponding percentiles for an XL-MS signal. These groups represent the misfolded structural ensemble that can generate the observed LiP-MS and/or XL-MS signals.

For each of the clusters 0 to 4, we choose a single structure that can generate the greatest number of experimental signals. If there is a tie (multiple structures generate the same number of consistent experiment signals), preference is given to the structure with the highest microstate probability, followed by the highest *Q* and *G* values.

### In silico temperature jump simulations and Arrhenius analysis of 4-diphosphocytidyl-2-*C*-methyl-d-erythritol kinase

To estimate the lifetime of the representative structure of each metastable state, we calculate the time it takes for this structure state to unfold. It is not feasible to trace the unfolding process of such large protein in conventional simulation; hence, we performed simulation at high temperatures, 600, 650, 700, 750, and 800 K, and then used Arrhenius analysis to extrapolate the unfolding at 298 K. The simulation protocol was similar to that used for ubiquitin and λ-repressor, with the exception that the salt concentration was set at 150 mM NaCl. For the production runs, at each temperature, we performed 50 independent trajectories in the NVT ensemble and then got the list of first-passage times that the structure is unfolded. To account for fluctuation, a trajectory was considered to reach the unfolded state if Q≤0.3 for 1 ns. The starting structures are highly native-like (*Q* > 0.7; see Fig. 3B in the main text), so there is an initial delay time during which native contacts are partially lost, but the system has not yet reached the unfolding threshold. This results in a delay time in the survival probability curves, during which the fraction of unfolded conformations remains zero (fig. S4). Once sufficient disruption of native contacts occurs, the survival probability begins to decay exponentially. The survival probability was then fitted to a single-exponential function to find the fitting parameters (*t*_0_ and *k*)

SU(t)={1,0≤t<t0e−k(t−t0),t≥t0 (10)

where *t*_0_ is the delay time of entanglement (fig. S4). The apparent unfolding rate at temperature *T* is $k_{\text{app}}\left(T\right)=\frac{1}{t_{0}+\frac{1}{k}}$.

The apparent unfolding rates are found to have a super-Arrhenius behavior (figs. S5 and S6) (*20*, *54*) and are then fitted as the function of *T*

$$\text{ln}\left(k_{\text{app}}\right)=\frac{a}{T^{2}}+\frac{b}{T}+c$$ (11)

Here *a*, *b*, and *c* are the free fitting parameters. We then extrapolated to obtain the apparent unfolding rate at the target temperature of 298 K. The extrapolated unfolding time in simulation is $\tau_{\text{sim}}=\frac{1}{k_{\text{app}}}$.

The uncertainties were estimated using bootstrapped resampling with 10^4^ iterations. For each iteration, at each temperature, we randomly selected with replacement 50 samples from the list of mean first-passage times of the original data at that temperature and then repeated the fitting procedure on the resampled data. Resampled datasets were discarded if the coefficient of determination $R^{2}<0.9$.

### Limited proteolysis experiments

To perform limited proteolysis, 2 μl of PK (from Tritirachium album) stock solutions (prepared at a concentration of 0.1 mg/ml in native buffer with 20% glycerol and then stored at −20°C for future use) was added to either native or refolded samples, which were prepared on a 200-μl scale. The reaction mixtures were rapidly mixed using a pipette [the enzyme:substrate ratio is 1:100 (w/w)]. Samples were incubated for 1 min before being transferred to a preheated mineral oil bath at 105°C to quench the PK activity. The samples were left in the oil bath for 5 min. After quenching, the proteolyzed samples were transferred to a microcentrifuge tube containing 152 mg of urea to reach a final concentration of 8 M. The limited proteolysis experiments were conducted in five replicates for both native and refolded IspE samples, resulting in a total of 10 samples.

### Cross-linking experiments

Two hundred microliters of native samples was prepared, consisting of 20 mM Hepes, 100 mM NaCl, 2 mM MgCl_2_, 1 mM tris(2-carboxyethyl)phosphine (TCEP), and 0.1% glycerol at pH 7.4, with IspE protein (0.1 mg/ml). Cross-linking was initiated by adding 2 μl of DSBU-*d*_0_ (100 mM stock in dimethyl sulfoxide; Thermo Fisher Scientific, A35459) to a final concentration of 1 mM. Simultaneously, refolded IspE samples were cross-linked with 2 μl of DSBU-*d*_12_ [deuterated disuccinimidyl dibutyric urea (DSBU); 100 mM stock in dimethyl sulfoxide]. DSBU-*d*_12_ was prepared as described in a separate publication (*55*).

The samples were then mixed and incubated at room temperature for 1 hour using a Roto-Mini rotator (Alkali Scientific RS3024) at 10 rpm. The reactions were quenched by adding 1 M tris-HCl (pH 7.5) to a final concentration of 20 mM, followed by 30-min incubation at room temperature, rotating at 10 rpm on the Roto-Mini. After quenching, the native and refolded samples were combined and transferred to a new microfuge tube containing 304 mg of urea to achieve a final concentration of 8 M. Cross-linking experiments were conducted in five replicates for both native and refolded IspE samples; after mixing the pairs, a total of five replicate samples (400 μl) was prepared.

### Modifying matched and nonaccessible cross-link score function

To computationally estimate the cross-linking propensity between residues 𝑖 and 𝑗, we first use the JWalk algorithm (*30*) to calculate the SASD between their C_α_ atoms. Subsequently, the cross-linking propensity is assessed using a customized version of the matched and nonaccessible cross-link (MNXL) score function (*30*). The original function was designed for pairs of lysines cross-linked by the dextran sulfate sodium linker (with a length of 11.4 Å); the MNXL score function needs adaptation for our XL-MS experiment where we use the DSBU cross-linker molecule (has a length of 12.5 Å) (*26*) and permit nonlysine residues to participate in cross-linking. To account for the differences, we have adjusted the parameters of the MNXL scoring function

MNXL score(SASDi,j)={N(SASDi,j,μi,j,σi,j2),SASDi,j≤thresholdi,j0,otherwise (12)

where $N\left({\text{SASD}}_{i,j},\mu_{i,j},\sigma_{i,j}^{2}\right)$ represents the normal distribution

$$N\left({\text{SASD}}_{i,j},\mu_{i,j},\sigma_{i,j}^{2}\right)=\frac{1}{\sqrt{2\pi }\sigma_{i,j}}e^{\frac{-1}{2}{\left(\frac{{\text{SASD}}_{i,j}-\mu_{i,j}}{\sigma_{i,j}}\right)}^{2}}$$ (13)

$\mu_{i,j}$ , $\sigma_{i,j}^{2}$ , and ${\text{threshold}}_{i,j}$ denote the mean, variance, and the SASD cutoff threshold for a specific pair of residues (i,j) involved in cross-linking, respectively. If the SASD of the pair of residues is less than the cutoff threshold, the propensity is determined by a normal distribution with the mean and variance specifically tailored for the pair of residues (table S6). If the SASD exceeds the threshold or if at least one residue in the pair is buried and inaccessible, then the propensity is assigned a value of 0.

### Comparison of misfolded and native clusters for consistency between simulations and experimental data

LiP-MS and XL-MS detected 13 and 20 structural signals, respectively, exhibiting significant differences between refolded and native samples. To assess the consistency between the misfolded ensembles from our simulations and experimental data, we examined whether the SASA of a region within five residues around the PK cut site identified from the experiment and the cross-linking propensity (MNXL score, Eq. 12) of pairs of residues from misfolded ensembles are statistically significantly different from the native ensemble from our simulations. The *P* values were computed using numerical permutation tests with 10^5^ iterations with the null hypothesis stating that there is no difference in the ensemble mean of SASA or cross-linking propensity between misfolded clusters and the native cluster. The *P* values were then adjusted using the Benjamini-Hochberg method (*29*). If adj.Pvalue≤0.05 , we reject the null hypothesis and accept the alternative hypothesis: The ensemble means of SASA or cross-linking propensity are significantly different between misfolded and native clusters. To mitigate the risk of underestimating the confidence interval of the ensemble mean (thus potentially overestimating the number of consistent signals) because of data correlation, we subsampled our data every 50 frames (as suggested by block averaging).

### Calculation of the relative aggregation propensity of the IspE misfolded clusters

The aggregation propensity of the misfolded clusters of IspE relative to the native cluster is quantified by the difference in the SASA of aggregation-prone regions between these two clusters. This propensity of misfolded cluster i relative to the native state is calculated using the following equation

$$\chi_{i}=\frac{{\langle \text{SASA}\rangle }_{i}-{\langle \text{SASA}\rangle }_{\text{native}}}{{\langle \text{SASA}\rangle }_{\text{native}}}\times 100\%$$ (14)

The aggregation-prone region was predicted by the AmylPred2 server (*31*). The uncertainties were estimated from bootstrap resampling with 10^5^ iterations.

### Uncertainty estimation

The uncertainties, represented as 95% confidence intervals, were estimated using bootstrapped resampling with 10^5^ iterations (except for the Arrhenius extrapolation, where 10^4^ iterations were used because of computational expensive). For a sample of *N* elements, in each iteration, we randomly selected *N* values from the original data with replacement. The mean was calculated from these resampled values, and the distribution of these means was used to estimate the 95% confidence interval.

### Permutation test

To evaluate whether the sample means ( $x_{A}$ and $x_{B}$ ) of two groups, A (with size *n*_A_) and B (with size *n*_B_), are significantly different, we performed a permutation test with 10^5^ iterations. The null hypothesis states that there is no difference between the sample means of the two groups. First, we calculated the absolute observed mean difference: $T_{\text{obs}}=|x_{A}-x_{B}\;|$ . Next, we pooled all the data from both groups and randomly selected, without replacement, *n*_A_ values for group A and *n*_B_ values for group B to form two new resampled groups. We then computed the absolute mean difference for these resampled groups. This process was repeated for 10^5^ iterations, generating a distribution of mean differences under the null hypothesis.

The two-tailed *P* value was calculated as the proportion of times the absolute resampled mean difference was equal or more extreme than the absolute observed difference ( $T_{\text{obs}}$ ). For multiple test comparison, we applied Benjamini-Hochberg correction to *P* values. If the adj.Pvalue≤0.05 , we reject the null hypothesis and accept the alternative hypothesis suggesting a statistically significant difference between the sample means of the two groups.

### Supporting methods for LiP-MS and XL-MS experiments

Additional methods related to LiP-MS and XL-MS experiments can be found in the Supplementary Materials.

## Supplementary Materials

### The PDF file includes:

Supplementary Text Figs. S1 to S7 Tables S1 to S6 Legends for data S1 and S2 References
