# Supplementary material for: Non-native entanglement protein misfolding observed in all-atom simulations and supported by experimental structural ensembles
Source: Sci Adv. 2025 Aug 8;11(32):eadt8974. doi: 10.1126/sciadv.adt8974 (PMC12333692; doi:10.1126/sciadv.adt8974)
Supplement: Supplementary file 1 — Supplementary Text Figs. S1 to S7 Tables S1 to S6 Legends for data S1 and S2 References [file sciadv.adt8974_sm.pdf]

Science Advances
AAAS

Supplementary Materials for

Non-native entanglement protein misfolding observed in all-atom simulations and supported by experimental structural ensembles

Quyen V. Vu *et al.*

Corresponding author: Stephen D. Fried, sdfried@jhu.edu; Edward P. O'Brien, epo2@psu.edu

Sci. Adv. **11**, eadt8974 (2025)
DOI: 10.1126/sciadv.adt8974

The PDF file includes:

Supplementary Text
Figs. S1 to S7
Tables S1 to S6
Legends for data S1 and S2
References

Other Supplementary Material for this manuscript includes the following:

Data S1 and S2

Supplementary Text

Clustering entanglement changes

To visualize entanglement changes in the simulated structures, we apply a hierarchical clustering technique proposed in ref (*55*) to group similar entanglement changes together. This approach allows us to obtain a smaller, representative set of entanglement changes that highlights the key structural variations.

IspE expression and purification

We introduced the gene encoding E. *coli* IspE, tagged with a C-terminal 6xHis, into the pET21(+) vector between the BamHI and HindIII restriction sites and ordered it from Twist Bioscience as a pre-cloned plasmid. The plasmids were propagated in NEB 10β cells (NEB C3019) as needed, and protein expression was carried out using the BL21(DE3) strain (NEB C2527H).

For a standard expression procedure, plasmids were introduced into chemically competent BL21(DE3) cells by heat shock (42°C for 40 sec in a water bath). The cells were then recovered in SOC media at 37°C for 60 min, plated on LB agar supplemented with 50 µg/mL ampicillin, and incubated overnight at 37°C. Selected colonies were used to inoculate 5 mL starter cultures of LB broth containing 50 µg/mL ampicillin, which were incubated overnight (~16 h) at 37°C with agitation at 220 rpm. The overnight cultures were then transferred into 1 L of terrific broth (TB) with 50 µg/mL ampicillin, starting with an OD_{600} of 0.05, and incubated at 37°C with agitation at 220 rpm. Induction was performed when the OD_{600} reached 0.4 by adding IPTG to a final concentration of 0.1 mM. After 4 hours of induction, cells were harvested by centrifugation (Eppendorf 5910R) at 4000 g for 15 min at 4°C in two 500 mL centrifuge tubes. The supernatants were removed, and the cell pellets were flash-frozen in liquid nitrogen before being stored at -20°C.

To purify the protein, the cell pellets were initially resuspended in 25 mL of lysis buffer containing 20 mM HEPES-NaOH at pH 7.4, 100 mM NaCl, 2 mM $MgCl_2$, and 20 mM imidazole-HCl at pH 8.0. Phenylmethylsulfonyl fluoride (PMSF) was introduced as a protease inhibitor to a final concentration of 1 mM, along with Deoxyribonuclease I (DNase I) at a final concentration of 0.1 mg/mL. The mixture was homogenized on a water-ice bath using a homogenizer (QSonica) set to 55% amplitude with a cycle of 8 seconds on and 8 seconds off, for a total of 15 minutes of on-time. After homogenization, the lysate was clarified by centrifugation at 16,000 g for 15 minutes at 4°C, followed by purification via Ni-NTA chromatography. The clarified lysate was mixed with 2 mL of Ni-NTA resin that had been pre-equilibrated with lysis buffer. This mixture was incubated for 1 hour at 4°C with gentle rocking, then allowed to flow through the resin in a vertically positioned column. The Ni-NTA resin was washed four times with 4 mL of wash buffer (20 mM HEPES-NaOH pH 7.4, 100 mM NaCl, 2 mM $MgCl_2$, 40 mM imidazole-HCl pH 8.0) and the protein was eluted with 3 mL of elution buffer (20 mM HEPES-NaOH pH 7.4, 100 mM NaCl, 2 mM $MgCl_2$, 300 mM imidazole-HCl pH 8.0), which was passed through the Ni-NTA bed twice for complete recovery.

The eluted proteins were assessed for purity using standard Tris-Tricine SDS-PAGE. A single band at the expected molecular weight was visualized with Coomassie staining, indicating that no additional purification steps were necessary. The eluted fractions were pooled and dialyzed overnight into native buffer (20 mM HEPES-NaOH pH 7.4, 100 mM NaCl, 2 mM $MgCl_2$) using G2 dialysis cassettes with a 10 kDa molecular weight cutoff (Fisher Scientific).

Protein concentration was determined using the BCA assay (Thermo Fisher) according to the manufacturer's instructions. Two protein stocks were then prepared, each with a final concentration of 10 mg/mL. Stock 1, intended for generating “native samples”, was prepared in native buffer with 10% (v/v) glycerol. Stock 2, designated for refolding reactions, was prepared in the native buffer without glycerol. Both stocks were aliquoted into 50 μL portions, flash frozen in liquid nitrogen, and stored at -80°C for future use.

Native and Refolded Sample Preparation.

To prepare refolding samples, 10 μL of freshly-thawed Stock 2 was transferred into an empty microfuge tube and then dried using a Vacufuge centrifugal concentrator (Eppendorf). Unfolding was initiated by adding 10 μL of a freshly-prepared solution containing 6 M guanidinium chloride (GdmCl) and 10 mM tris(2-carboxyethyl)phosphine (TCEP) to the dried protein. The mixture was thoroughly mixed by pipetting, then split into five microfuge tubes, each containing 2 μL, and incubated at room temperature for 24 hr.

The following day, the refolding reaction was initiated by a 100-fold dilution, adding 198 μL of refolding dilution buffer (20 mM HEPES-NaOH pH 7.4, 100 mM NaCl, 2 mM $MgCl_2$, 0.91 mM TCEP, 0.1% (v/v) glycerol) to 2 μL of the unfolded samples, resulting in a final protein concentration of 0.1 mg/mL. The samples were then incubated at room temperature for 1 hr to allow refolding, with the reactions performed in five replicates.

To prepare native samples, which did not go through the unfolding-refolding process, 2 μL of Stock 1 was diluted 100-fold by adding 198 μL of native dilution buffer (20 mM HEPES-NaOH pH 7.4, 100 mM NaCl, 2 mM $MgCl_2$, 1.01 mM TCEP, 0.06 M GdmCl), with five replicates prepared, resulting in a final concentration of 0.1 mg/mL. After dilution, the final protein concentration in both the native and refolded samples was the same (0.1 mg/mL), with consistent concentrations of salts and osmolytes across all samples.

It was observed that the refolding reactions led to detectable precipitation. However, the aggregated protein pellets could be redissolved by simple pipette trituration (fig. S7), leading to the inference that these aggregates are not stable. Hence, limited proteolysis and crosslinking experiments were carried out on the triturated refolded samples and the native sample without further fractionation.

Mass Spectrometry Sample Preparation.

A freshly prepared 1 M stock of DTT was added to each crosslinking and limited proteolysis sample to a final concentration of 10 mM. The samples were incubated at 37°C for 30 min with agitation on a thermomixer. Freshly prepared 1 M iodoacetamide (IAA) was then added to a final concentration of 40 mM to alkylate the thiol groups, and the samples were incubated at room temperature in the dark for 45 minutes.

For the crosslinking samples, digestion with Lys-C (NEB P8109S) was initiated by adding 4 μL of a 0.1 mg/mL Lys-C stock (1:100 w/w ratio with respect to the substrate). These samples were incubated at 37°C and 700 rpm for 1.5 hr in a thermomixer. Following alkylation for limited proteolysis samples or Lys-C digestion for crosslinking samples, the solutions were diluted 4-fold by adding 100 mM ammonium bicarbonate, pH 8, to bring the final urea concentration down to 2 M. Trypsin (0.1 mg/mL stock, NEB P8101S, 1:50 w/w ratio with respect to the substrate) was added to the samples—4 μL for limited proteolysis samples and 8 μL for crosslinking samples. The samples were then digested overnight at 25°C with agitation at 700 rpm in a thermomixer.

After digestion, the peptides were acidified with trifluoroacetic acid (TFA, Acros) to a final concentration of 1% (v/v). Desalting of the peptide samples was performed using Sep-Pak C18

1cc Vac Cartridges (Waters). Cartridges were preconditioned with 2 mL of buffer B (80% acetonitrile, 0.5% TFA in LC-MS grade water) and equilibrated with 4 mL of buffer A (0.5% TFA in LC-MS grade water) before loading the sample slowly under a diminished vacuum. The acidified peptides were then loaded onto the cartridges under vacuum, washed with 4 mL of buffer A, and eluted with 1 mL of buffer B. During elution, cartridges were suspended above 15 mL conical tubes, placed in a swing-bucket rotor (Eppendorf 5910R), and spun for 2 min at 300 rpm. Eluted peptides were transferred from Falcon tubes back into microfuge tubes and dried using a Vacufuge centrifugal concentrator (Eppendorf) and stored at -80°C until mass spectrometry analysis.

LC-MS/MS Acquisition.

A Thermo Q-Exactive HF-X Orbitrap mass spectrometer, coupled with a Thermo UltiMate 3000 UHPLC system and an Acclaim Pepmap RSLC C18 column (75 µm × 25 cm, 2 µm, 100 Å), was used to analyze all samples.

Approximately 1 µg of protein was injected on to the column. The column temperature was set to 40°C and the flow rate was maintained at 300 nL/min for the duration of the run. Solvent A consist of 0.1% formic acid in LC-MS grade water, and solvent B consist 0.1% formic acid in acetonitrile. After accumulation of peptides onto the trap column for 10 min (during which the column was held at 2% solvent B), a gradient of 80 minutes was applied for limited proteolysis samples and 200 minutes for crosslinking samples.

For the limited proteolysis experiment, samples were processed as follows. Peptides were eluted through the separation column using a rapid gradient increase from 2% B to 5% B over 5 minutes. This was followed by a 47 min linear gradient from 5% B to 25% B. The gradient was then increased from 25% B to 40% B over 13 min, followed by a further rise to 90% B over the next 5 minutes. Finally, the column was cleaned using a sawtooth gradient to remove any remaining peptides between runs, over a period of 10 minutes.

Data acquisition was performed using a full MS scan followed by a data-dependent top 20 strategy in positive ion mode. Full MS scan data were collected across the m/z range of 350-1500, with a resolution of 120,000 at m/z 200, an automated gain control (AGC) target of 3×10^6, and a maximum injection time of 64 ms. The data-dependent scans were captured at a resolution of 30,000 at m/z 200, with an AGC target of 1×10^5, a maximum injection time of 55 ms, and a 1.4 m/z isolation window. Peptides were subjected to high-energy collisional dissociation (HCD) with a normalized collision energy of 27, followed by reanalysis via MS2. Fragments with charges of 1 and above 6 were excluded from the analysis, and a dynamic exclusion window of 30 sec was applied during the data-dependent scans.

For the crosslinking samples, peptides were eluted from the separation column using a 40-min linear gradient that started at 2% B and increased to 5% B. The gradient was then raised to 30% B over the next 120 min, followed by an increase to 40% B over the subsequent 25 min, and finally elevated to 90% B over the next 5 min. To ensure removal of any residual peptides between runs, the column was cleaned with a 10 min sawtooth gradient.

Data acquisition was conducted in positive ion mode, beginning with a full MS scan followed by a data-dependent top 20 strategy. Full MS scan data were acquired across an m/z range of 300-1800, with a resolution of 120,000 at m/z 200, an AGC target of 3×10^6, and a maximum injection time of 100 ms. Data-dependent scans were collected at a resolution of 60,000 at m/z 200, with an AGC target of 2×10^5, a maximum injection time of 250 ms, and a 2 m/z isolation window. These scans were triggered by twin signals reflecting the d_0-d_{12} mass difference of 12.075 Da. Fragments

with charges of 1, 2, and greater than 8 were excluded from analysis, and a dynamic exclusion window of 60 sec was applied. Peptides underwent high-energy collisional dissociation (HCD) with stepped normalized collision energies (NCEs of 22, 25, and 28) before being reanalyzed by MS/MS.

Data Analysis for Limited Proteolysis Experiments.

We used FragPipe (*62*) (V21.1) for spectral searches and label-free quantification (LFQ) of the peptides identified. The LFQ analysis was carried out on five replicates each for native control and refolded samples. Peptide identification was conducted using the MsFragger node, which was set up for a semi-tryptic search, allowing a maximum of 2 missed cleavages. At the MS1 level, the precursor mass tolerance was defined at 10 ppm, and at the MS/MS level, the fragment ion tolerance was established at 20 ppm. The search included methionine oxidation and N-terminal acetylation as variable modifications, while cysteine carbamidomethylation was treated as a fixed modification. The analysis was performed against a FASTA file comprising the complete *E. coli* proteome sequence (UP000000625), alongside decoy sequences and potential contaminants.

These data were further analyzed using FLiPPR (*63*), which is available on GitHub (https://github.com/FriedLabJHU/FragPipe-Limited-Proteolysis-Processor). In summary, normalized ion counts were compiled across the refolded and native replicates for each successfully identified peptide group. Effect sizes were calculated as the ratio of the averages, reported on a $\log_2$ scale, while p-values were reported on a $-\log_{10}$ scale, assessed using t-tests with Welch's correction for unequal variances between populations. Special handling was applied to missing data. If an ion was detected in all five replicates of either the native or refolded samples but not detected in any of the other five injections, Gaussian imputation was used to fill the missing values, classifying the peptide as an "all-or-nothing" peptide. In cases where a single value was missing across the ten injections, that single value was excluded, and effect sizes and p-values were calculated from the remaining nine values. If more than one value was missing in any other combination, the data for that ion was discarded. Ions corresponding to a common cut-site are merged together and assessed for consistency. Multiple hypothesis correction was applied using the Benjamini-Hochberg procedure. Peptides were considered to exhibit a significant change in proteolytic susceptibility between the refolded and native forms if they showed at least a 2-fold difference in abundance $\left(\left|\log_2(\frac{\text{refolded}}{\text{native}})\right| \geq 1\right)$ and admitted an $\text{adj. p} - \text{value} \leq 0.05$, or $-\log_{10}\text{adj. p} - \text{value} \geq 1.3$). Effect sizes and p-values for each cut-site are reported in Table 2 and Data S1.

Data Analysis for Crosslinking Experiments.

The Proteome Discoverer Software Suite (PD, v2.4, Thermo Fisher) was utilized, incorporating the Minora feature detector algorithm, to perform spectral searches and label-free quantification (LFQ). The LFQ analysis was conducted across the five replicate crosslinked raw files. Peptide identification was carried out using the MSFragger node, configured for a standard-tryptic search, allowing up to 2 missed cleavages. The precursor mass tolerance was set to 10 ppm at the MS1 level, while a fragment ion tolerance of 0.02 Da was applied at the MS/MS level. Oxidation of methionine and acetylation of the N-terminus were allowed as dynamic modifications, with carbamidomethylation on cysteines established as a static modification. The search was conducted using a FASTA file containing only the sequence for *E. coli* IspE (P62615).

Although PD is not used to identify crosslinked peptides, it generates an output known as a consensus feature file. This file contains the detected ion intensities for every ion, annotated by its

precursor m/z and retention time, regardless of whether they are linked to a peptide-spectrum match (PSM) or not. We utilized these consensus feature outputs to extract ion intensities for crosslinked peptides that were confidently matched to MS2 spectra using XiSearch (*64*).

To identify crosslinks using XiSearch, the raw data were first converted to .mgf files and recalibrated with MSConvert. The standard "small-scale" setting for searching DSBU crosslinks was applied. Crosslink specificity sites were defined as lysine and protein N-termini for site1, and lysines, protein N-termini, serines, threonines, and tyrosines for site2. Methionine oxidation was treated as a variable modification, while cysteine carbamidomethylation was enforced as a fixed modification. XiSearch was configured to search only for b- and y-ions, with trypsin as the digestion enzyme. Both water loss and ammonia loss were permitted. The settings allowed for up to three missed cleavages and two modifications per peptide, with mass deviations permitted up to 5 ppm for MS1 and 10 ppm for MS/MS.

To run XiSearch, the settings file described above was used, along with the .mgf files and a FASTA file containing only the sequence for *E. coli* IspE (P62615). A second XiSearch was conducted using the same settings but with the crosslinker adjusted from standard DSBU to DSBU-d_{12}. In the settings file, DSBU-d_{12} was incorporated under advanced configuration by providing the crosslinker's name and mass (208.15979231 Da, with a mass difference of 12.075 Da from DSBU-d_0). After processing the data with XiSearch, the output files from the DSBU-d_0 and DSBU-d_{12} searches were provided into XiFDR, along with the XiSearch settings file and the FASTA file. XiFDR was run with its default settings, with one modification: a prefilter was applied to ensure the doublet count (CCfragmentdoubletcount) was greater than 0. Finally, the output files from XiFDR for DSBU-d_0 and DSBU-d_{12} were concatenated.

To process the data, including the assignments from XiSearch/XiFDR and the quantifications from PD consensus feature files, we utilized custom Python scripts, which are available upon request. The workflow begins with a list of confidently identified crosslinked peptide-spectrum matches (XSMs) from XiSearch/XiFDR. Each crosslinked peptide is annotated with details such as the injection source, scan ID, retention time, and precursor m/z. Using this information, we map the crosslinked peptide precursor to the corresponding feature in the PD consensus feature file, based on m/z and retention time. This mapping yields five ion intensities for each crosslinked peptide across the five replicates. If any values are missing, they are imputed with an ion count of 10^3, representing an estimated detection limit.

If the crosslinked peptide was crosslinked with DSBU-d_0, the script attempts to identify a corresponding feature for the same species crosslinked with DSBU-d_{12}. To do this, it searches for a feature with a matching retention time (within a 3 min tolerance) and a precursor m/z that is higher by 12.075 divided by the charge state (within a 10 ppm tolerance). If no matching consensus feature is found, the heavy crosslinked peptide is considered absent, and its values are filled with an imputed ion count of 10^3. However, if a matching consensus feature is identified, the corresponding ion intensities are extracted and combined with those of the DSBU-d_0 replicates. For crosslinked peptides that were originally crosslinked with DSBU-d_{12}, the script performs the same process. It looks for a corresponding feature that would correspond to the same species crosslinked with DSBU-d_0 with a matching retention time (±3 min) and a precursor m/z that is lower by 12.075 divided by the charge state (±10 ppm).

We apply a filtering approach similar to the one used for limited proteolysis to these quantified crosslinked peptides. Specifically, a crosslinked peptide is retained if it has no missing values (i.e., five light ion intensities and five heavy ion intensities); or if a crosslinked peptide has five non-zero values corresponding to the refolded (or native) replicates and five imputed values

(set at 1000 ion counts) corresponding to the native (or refolded) replicates, it is classified as an "all-or-nothing" peptide and is also retained for further analysis. We then calculated the ratio for each consensus feature by dividing the total extracted ion intensities of the native samples by those of the refolded samples. P-values were determined using a t-test with Welch's correction for unequal population variances. Generally, a two-tailed t-test was applied, except in the "all-or-nothing" cases, where a one-tailed t-test was used.

Each unique crosslink, represented as a residue pair, could be detected as multiple distinct ions. This can occur when different peptides are crosslinked at the same two residues, or when the same crosslinked peptide is observed in various charge states or methionine oxidation states. To provide a single quantification for each unique crosslink, we aggregate the data from all ions that correspond to the same crosslink. If the ratios for the various ions show conflicting signs—for instance, if a crosslinked peptide is more abundant in the native sample for one charge state but more abundant in the refolded sample for another charge state—the crosslink is excluded from analysis. A "majority rules" approach is employed: if two ions agree on the sign while one does not, the feature with the conflicting result is discarded, and the two agreeing features are retained. For the remaining features, effect sizes are combined by calculating the median, and p-values are aggregated using Fisher's method. The Benjamini-Hochberg procedure is then applied to these combined p-values to compute adjusted p-values. Effect sizes and p-values for each unique crosslink are reported in Table 3 and Data S1.

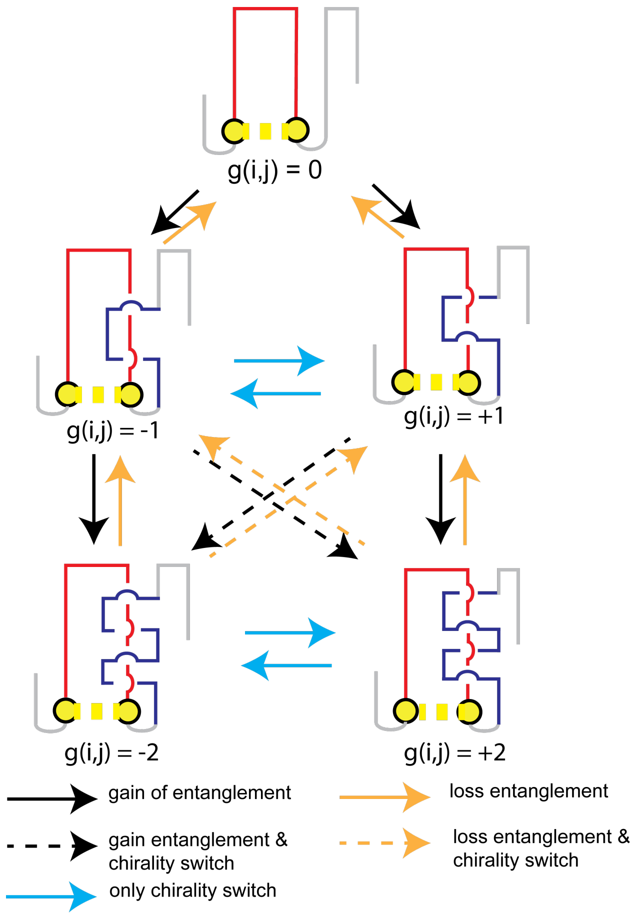

Fig. S1.

Schematic of different changes in topological entanglement observed with the Gaussian linker integration method. The closed loop is colored in red and the threading segment is in blue. The loop is closed by a non-covalent contact between two residues (yellow). The total linking number $g(i,j)$ of contact present at residues $(i,\ j)$ provides information on the topological entanglement between a loop formed by a non-covalent contact and the flanking terminal thread.

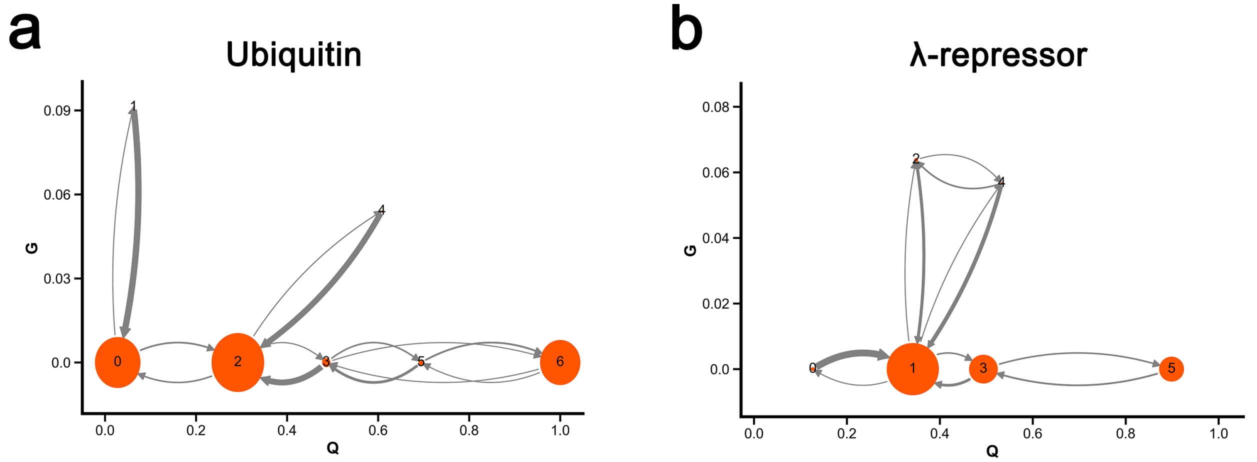

Fig. S2.

Structural network transition of Ubiquitin (a) and λ-repressor (b). The structural clusters were obtained through clustering analysis of the protein conformations, based on (Q, G) values. The arrows connecting the clusters represent transitions between them, and the width of each arrow is proportional to the number of observed transitions between the corresponding clusters.

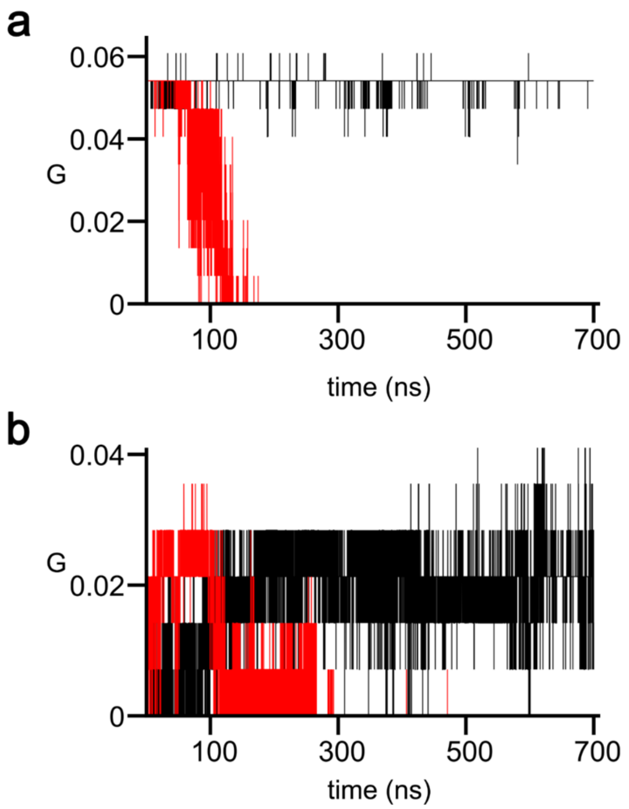

Fig. S3.

Time evolution of the G parameter starting from entangled conformations of Ubiquitin and λ-repressor. (a) Ubiquitin's G versus time for two trajectories, one trajectory does not disentangle up to 700 ns (black line) and the other trajectory disentangles at a time less than 700 ns (red line). (b) Same as Fig. S3a, but for λ-repressor.

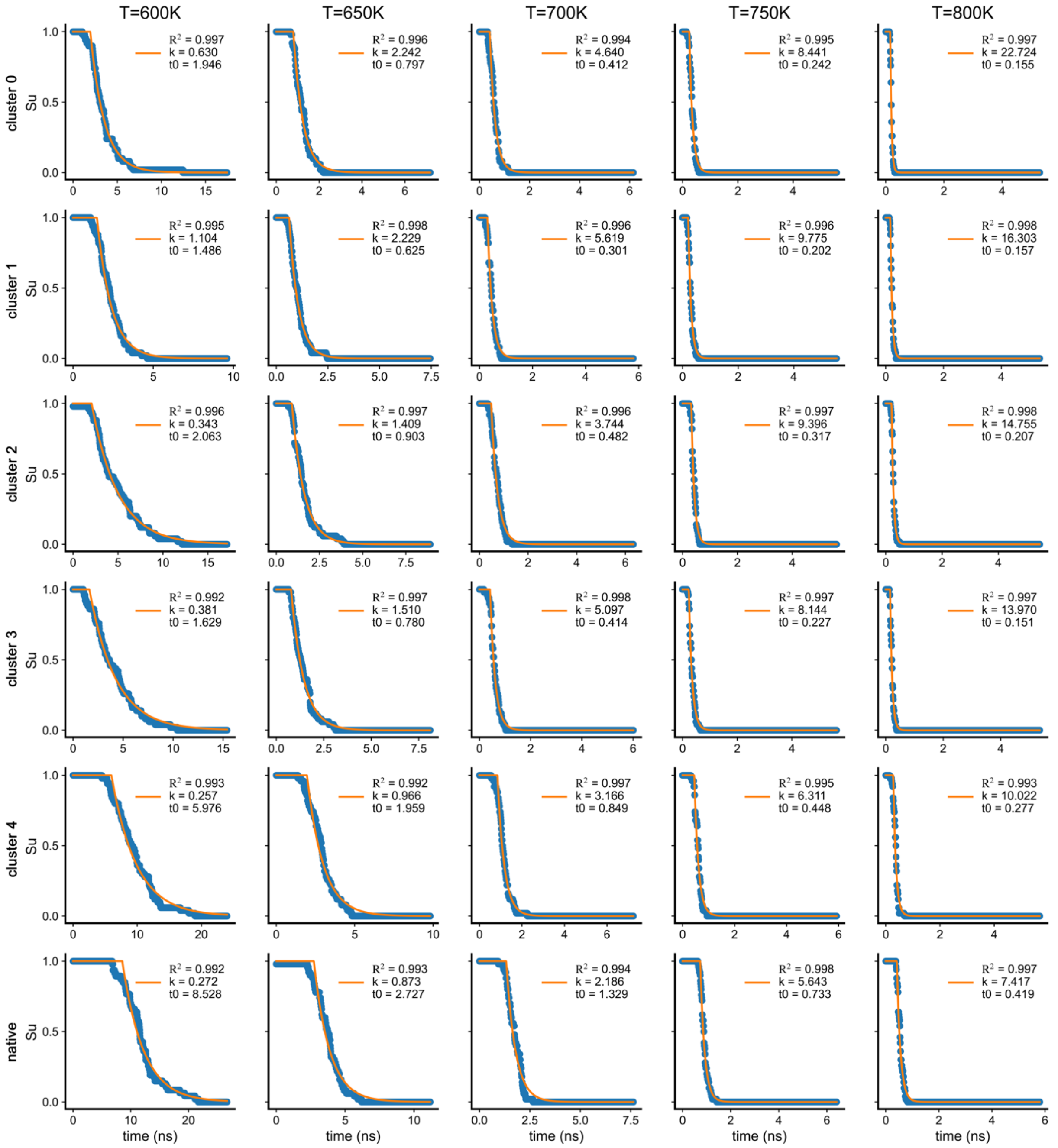

Fig. S4.

IspE Survival probability fitting. Survival probabilities of structure from misfolded clusters and native cluster vs. time at different simulation temperatures that were fitted by an exponential function with delay time (orange). The coefficient of determination R^2 and fitting parameters (unfolding rate k and delay time t_0) are presented in the legends.

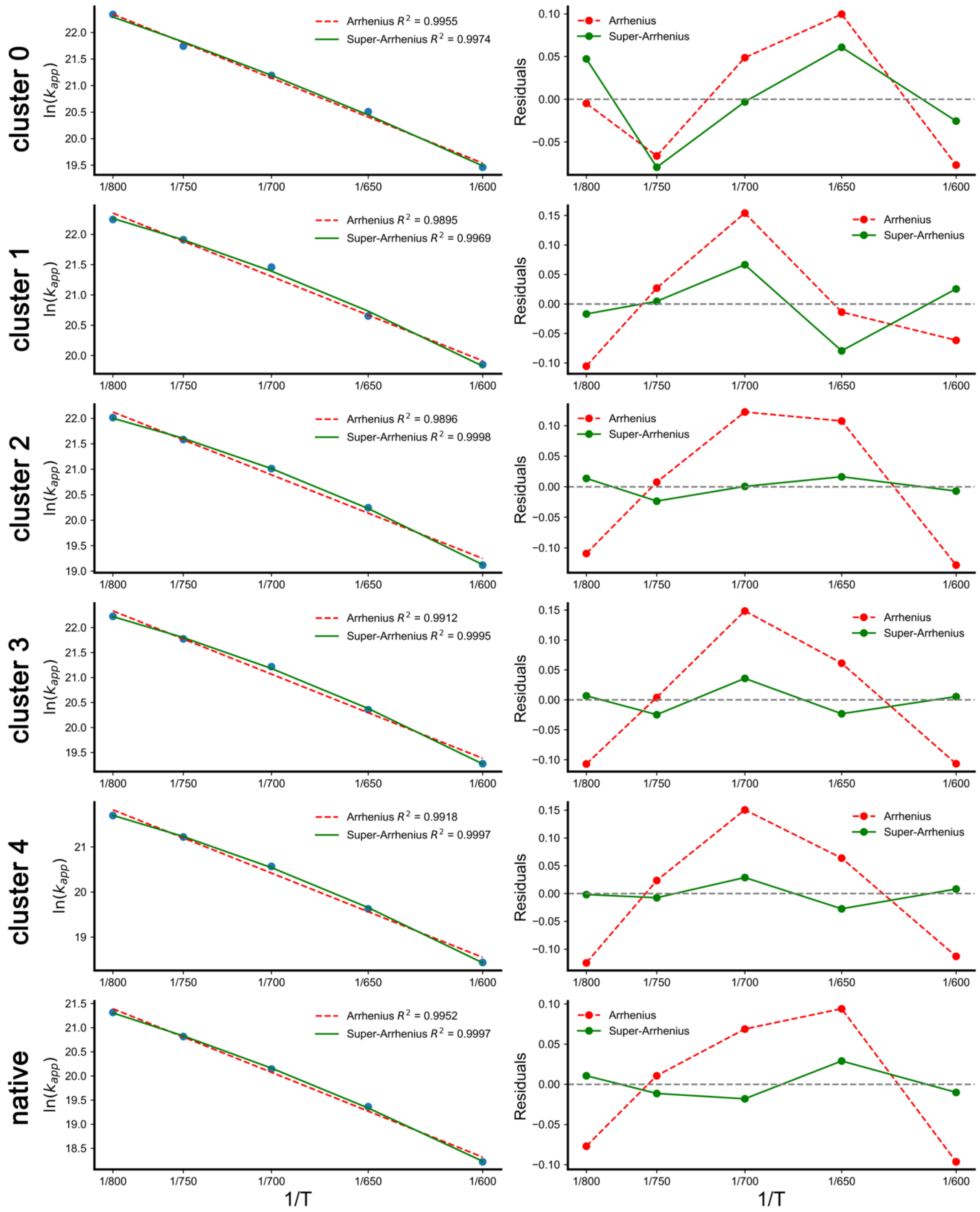

Fig. S5.

Comparison of Arrhenius and super-Arrhenius fits to the temperature dependence of $\ln K_{app}$. The left column shows the coefficient of determination R^2, while the right column displays the residuals. The residuals for the native cluster, and clusters 1, 2, 3 and 4 are non-

random for the Arrhenius model, and random for the super-Arrhenius model. Cluster 0 shows non-random residuals for both models. This demonstrates that in almost all cases the super-Arrhenius model is the appropriate model.

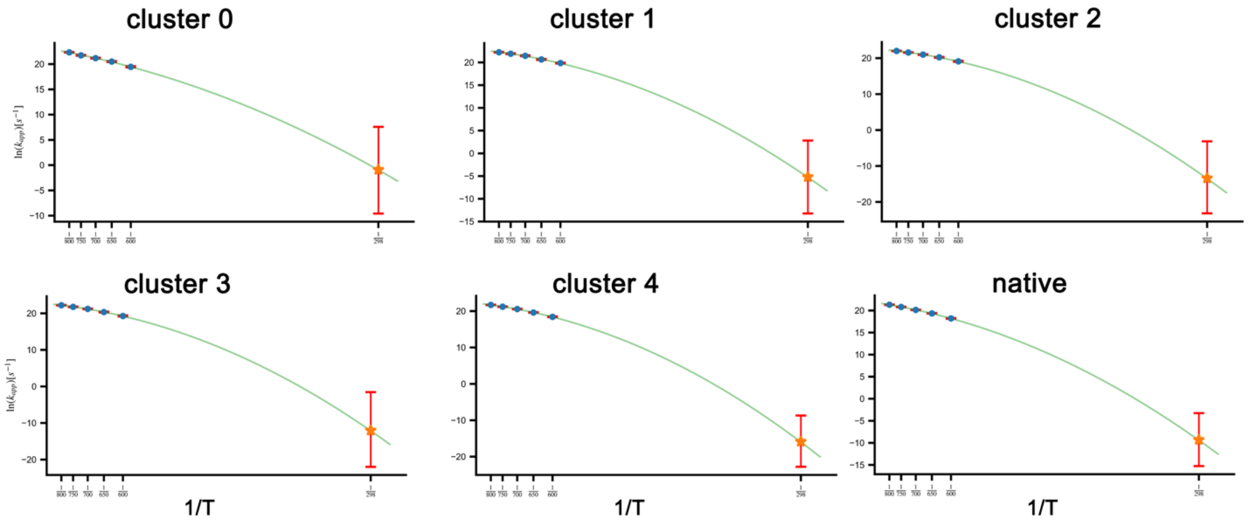

Fig. S6.

Extrapolation of ln K_{app} at 298K. For each cluster, the apparent unfolding rate at 298K (orange star symbol) is extrapolated by fitting the temperature dependence of ln K_{app} using a super-Arrhenius model. Error bars represent the 95% confident intervals.

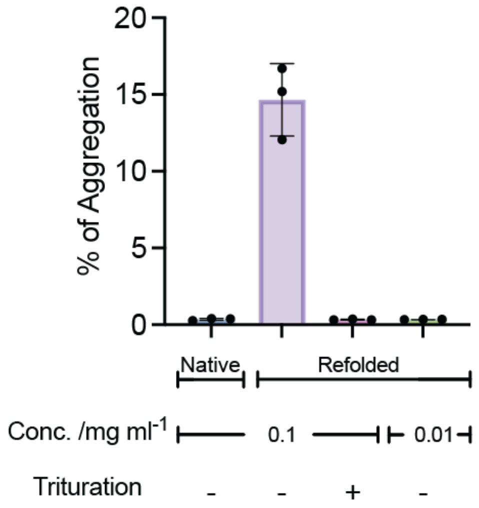

Fig. S7.

IspE aggregation assay. Bar chart shows the quantification of protein aggregation in native and refolded samples at two concentrations of IspE, 0.1 mg/mL and 0.01 mg/mL, 1 hr after refolding conditions were established. Protein precipitation was detected upon refolding; however, the aggregation was not detectable after trituration.

Table S1.

Definitions of the different types of change in entanglement that are possible.

**Type**	**Change in entanglement**	**Change in chirality**	**Conditions**
G_0	Gain	No	$\lvert g^{current}(i,j)\rvert > \lvert g^{native}(i,j)\rvert$ *and* $g^{current}(i,j) \times g^{native}(i,j) \geq 0$
G_1	Gain	Yes	$\lvert g^{current}(i,j)\rvert > \lvert g^{native}(i,j)\rvert$ *and* $g^{current}(i,j) \times g^{native}(i,j) < 0$
G_2	Lose	No	$\lvert g^{current}(i,j)\rvert < \lvert g^{native}(i,j)\rvert$ *and* $g^{current}(i,j) \times g^{native}(i,j) \geq 0$
G_3	Lose	Yes	$\lvert g^{current}(i,j)\rvert < \lvert g^{native}(i,j)\rvert$ *and* $g^{current}(i,j) \times g^{native}(i,j) < 0$
G_4	None	Yes	$\lvert g^{current}(i,j)\rvert = \lvert g^{native}(i,j)\rvert$ *and* $g^{current}(i,j) \times g^{native}(i,j) < 0$
G_5	None	None	$\lvert g^{current}(i,j)\rvert = \lvert g^{native}(i,j)\rvert$ *and* $g^{current}(i,j) \times g^{native}(i,j) \geq 0$

Table S2.

Properties of entangled states of Ubiquitin and λ-repressor. The conformational ID was indexed by sorting the degree of entanglement G in descending order for each protein.

ID (1)	Fraction of native contacts Q (2)	Degree of entanglement G (3)	Residues that close the loop and crossing residues (4)	Secondary structure similarity (5)	Persistence Time (ns) (6)
**Ubiquitin**					
1	0.61	0.054	[45-67], [5,13]	0.71	700, 700, 520
2	0.64	0.054	[45-67], [5,13]	0.60	215, 700, 553
3	0.63	0.054	[45-68], [5,13]	0.57	700, 700, 481
4	0.61	0.054	[44-69], [5,13]	0.43	700, 700, 700
5	0.61	0.054	[44-70], [5,12]	0.33	700, 337, 700
6	0.63	0.054	[45-67], [5,13]	0.69	700, 163, 150
7	0.61	0.054	[44-67], [5,13]	0.64	700, 700, 627
8	0.61	0.054	[44-70], [5,12]	0.48	700, 700, 700
9	0.63	0.054	[45-67], [5,13]	0.52	700, 700, 578
10	0.61	0.054	[45-67], [5,13]	0.55	700, 700, 700
11	0.63	0.054	[45-67], [5,13]	0.57	700, 700, 700
12	0.63	0.054	[45-67], [5,13]	0.60	700, 464, 666
13	0.61	0.054	[45-68], [5,12]	0.50	700, 121,700
14	0.63	0.054	[45-67], [5,13]	0.48	700, 700, 700
15	0.63	0.054	[45-68], [5,13]	0.55	700, 700, 700
16	0.61	0.054	[44-70], [5,13]	0.60	700, 700, 700
17	0.63	0.054	[44-70], [5,13]	0.48	700, 700, 700
18	0.61	0.054	[45-67], [5,12]	0.50	700, 700, 700
19	0.63	0.054	[45-68], [5,13]	0.57	700, 700, 700
20	0.63	0.047	[45-67], [4,13]	0.19	40, 100, 55
21	0.74	0.034	[23-53], [66]	0.81	0, 0, 0
**λ-repressor**					
22	0.62	0.028	[57-75], [9]	0.71	1, 22, 1
23	0.61	0.028	[57-75], [11]	0.71	12, 18, 615
24	0.62	0.028	[57-75], [11]	0.73	1, 2, 25
25	0.61	0.021	[57-75], [12]	0.64	2, 700, 523
26	0.65	0.021	[57-75], [11]	0.75	700, 0, 182
27	0.62	0.021	[54-75], [12]	0.73	1, 19, 2
28	0.61	0.021	[53-75], [11]	0.73	178, 700, 200
29	0.61	0.014	[57-75], [12]	0.68	2, 3, 18
30	0.63	0.014	[57-75], [11]	0.73	106, 67, 603
31	0.65	0.007	[57-75], [12]	0.73	0, 217, 0
32	0.63	0.007	[57-75], [12]	0.71	700, 178, 0

33	0.66	0.007	[54-75], [11]	0.71	700, 700, 10

(1) Entangled state ID used in this study.

(2) Fraction of native contacts, we only selected entangled structures that had at least 60% of their native contacts formed as the starting structures for the subsequent all-atom simulations.

(3) Degree of entanglement G, is the fraction of native contacts with changes in entanglement calculated by Eq. 4.

(4) Representative native contact that closes the loop (first square brackets) and list of crossing residues (second square brackets). Note well, some entanglements have a threading segment that pierces the loop once (hence a single crossing residue is reported), while in others the threading segment pierces the loop twice (hence two crossing residues are reported).

(5) Secondary structure similarity is defined as the fraction of residues that are in their native secondary structure in the current structure.

(6) Time it takes for the non-native entanglement to disentangle. 3 replicas were run for each entangled state found from D.E. Shaw's protein folding trajectories. Note well that the simulations were only run for 700 ns. Therefore, the entries of 700 ns mean the entanglement state formed the entire simulation time.

Table S3.
Radius of gyration and solubility of the native and entangled states of Ubiquitin, λ-repressor.

ID	Radius of gyration (R_g, nm)	R_g difference compared to native structure (%)	Solubility
**Ubiquitin**			
Native Structure	1.18		
1	1.24	5.3%	0.836
2	1.19	0.9%	0.915
3	1.21	2.6%	0.937
4	1.30	10.6%	0.840
5	1.23	4.3%	0.897
6	1.25	6.6%	0.928
7	1.23	4.8%	0.926
8	1.23	5.1%	0.849
9	1.25	6.2%	0.946
10	1.24	5.5%	0.908
11	1.21	3.1%	0.903
12	1.20	1.7%	0.940
13	1.20	2.2%	0.990
14	1.20	2.0%	0.955
15	1.22	3.6%	0.903
16	1.30	10.8%	0.901
17	1.26	7.3%	0.963
18	1.26	6.8%	0.787
19	1.26	7.1%	0.958
20	1.31	11.2%	0.976
21	1.27	7.7%	0.807
The average difference between entangled states and native structure		5.5%	
**λ-repressor**			
Native Structure	1.19		
22	1.25	5.7%	0.802
23	1.25	5.4%	0.832
24	1.21	2.2%	0.770
25	1.25	5.3%	0.739
26	1.27	7.5%	0.747
27	1.27	7.3%	0.755
28	1.24	4.9%	0.729
29	1.25	5.8%	0.673
30	1.21	2.0%	0.766

31	1.26	6.1%	0.710
32	1.26	6.1%	0.719
33	1.24	4.4%	0.765
The average difference between entangled states and native structure		5.2%	

The solubility of the entangled states was calculated using Eq. 7.

Table S4.
Entangled state ID (Table S2) and the frame corresponding to DE Shaw's trajectories.

ID	Trajectory's names are from the D. E Shaw group as reported in Ref. (12, 13)	Simulation Frame
Ubiquitin		
1	pnas2013-unfold-2-c-alpha-000.dcd	49551
2		49552
3		49553
4		49601
5		49548
6		49517
7		49519
8		49544
9		49599
10		49651
11		49576
12		49577
13		49578
14		49579
15		49582
16		49593
17		49595
18		49570
19		49594
20	pnas2013-native-4-c-alpha-006.dcd	13525
21	pnas2013-native-5-c-alpha-002.dcd	48392
λ-repressor		
22	lambda-0-c-alpha-001.dcd	54713
23		54729
24		59891
25		59863
26		59878
27		59698
28		59884
29		59870
30		59890
31		59861
32		59866

33		59883

Table S5.

Representative Entanglements in the IspE Crystal Structure.

Entanglement ID	Residues that close the loop	Crossing Residues	g_N, g_C
1	[26, 185]	[8, 10]	-1.68, -0.54
2	[32, 141]	[8]	-0.77, 0.02

Table S6.
Amino acid dependent parameters (in unit Å) in crosslinking propensity score.

(i, j)	$\mu_{i,j}$(Å)	$\sigma_{i,j}$(Å)	$\text{threshold}_{i,j}$(Å)
(K, K)	19.7	6.4	34.1
(K, S)	15.9	5.1	30.3
(K, T)	15.9	5.1	30.3
(K, Y)	19.9	6.4	34.3

Data S1. (separate file)

LiP-MS, XL-MS data and comparison between misfolded and native clusters from simulations based on experimental signals.

Data S2. (separate file)

Ten structures from each misfolded cluster of IspE in PDB format.

REFERENCES AND NOTES

1. Y. Jiang, S. S. Neti, I. Sitarik, P. Pradhan, P. To, Y. Xia, S. D. Fried, S. J. Booker, E. P. O'Brien, How synonymous mutations alter enzyme structure and function over long timescales. *Nat. Chem.* **15**, 308–318 (2023).

2. D. A. Nissley, Y. Jiang, F. Trovato, I. Sitarik, K. B. Narayan, P. To, Y. Xia, S. D. Fried, E. P. O'Brien, Universal protein misfolding intermediates can bypass the proteostasis network and remain soluble and less functional. *Nat. Commun.* **13**, 3081 (2022).

3. R. Halder, D. A. Nissley, I. Sitarik, Y. Jiang, Y. Rao, Q. V. Vu, M. S. Li, J. Pritchard, E. P. O'Brien, How soluble misfolded proteins bypass chaperones at the molecular level. *Nat. Commun.* **14**, 3689 (2023).

4. M. Baiesi, E. Orlandini, A. Trovato, F. Seno, Linking in domain-swapped protein dimers. *Sci. Rep.* **6**, 33872 (2016).

5. M. Baiesi, E. Orlandini, F. Seno, A. Trovato, Exploring the correlation between the folding rates of proteins and the entanglement of their native states. *J. Phys. A. Math. Theor.* **50**, 504001 (2017).

6. M. Baiesi, E. Orlandini, F. Seno, A. Trovato, Sequence and structural patterns detected in entangled proteins reveal the importance of co-translational folding. *Sci. Rep.* **9**, 8426 (2019).

7. V. Rana, I. Sitarik, J. Petucci, Y. Jiang, H. Song, E. P. O'Brien, Non-covalent lasso entanglements in folded proteins: Prevalence, functional implications, and evolutionary significance. *J. Mol. Biol.* **436**, 168459 (2024).

8. A. A. Komar, T. Lesnik, C. Reiss, Synonymous codon substitutions affect ribosome traffic and protein folding during in vitro translation. *FEBS Lett.* **462**, 387–391 (1999).

9. M. Zhou, J. Guo, J. Cha, M. Chae, S. Chen, J. M. Barral, M. S. Sachs, Y. Liu, Non-optimal codon usage affects expression, structure and function of clock protein FRQ. *Nature* **494**, 111–115 (2013).

10. M. Zhou, T. Wang, J. Fu, G. Xiao, Y. Liu, Nonoptimal codon usage influences protein structure in intrinsically disordered regions. *Mol. Microbiol.* **97**, 974–987 (2015).

11. D. A. Nissley, Q. V. Vu, F. Trovato, N. Ahmed, Y. Jiang, M. S. Li, E. P. O'Brien, Electrostatic interactions govern extreme nascent protein ejection times from ribosomes and can delay ribosome recycling. *J. Am. Chem. Soc.* **142**, 6103–6110 (2020).

12. S. Piana, K. Lindorff-Larsen, D. E. Shaw, Atomic-level description of ubiquitin folding. *Proc. Natl. Acad. Sci. U.S.A.* **110**, 5915–5920 (2013).

13. K. Lindorff-Larsen, S. Piana, R. O. Dror, D. E. Shaw, How fast-folding proteins fold. *Science* **334**, 517–520 (2011).

14. T. Sivaraman, C. B. Arrington, A. D. Robertson, Kinetics of unfolding and folding from amide hydrogen exchange in native ubiquitin. *Nat. Struct. Biol.* **8**, 331–333 (2001).

15. W. Y. Yang, M. Gruebele, Folding at the speed limit. *Nature* **423**, 193–197 (2003).

16. D. Frishman, P. Argos, Knowledge-based protein secondary structure assignment. *Proteins Struc. Func. Genet.* **23**, 566–579 (1995).

17. K. L. Maxwell, D. Wildes, A. Zarrine-Afsar, M. A. De Los Rios, A. G. Brown, C. T. Friel, L. Hedberg, J.-C. Horng, D. Bona, E. J. Miller, A. Vallée-Bélisle, E. R. G. Main, F. Bemporad, L. Qiu, K. Teilum, N.-D. Vu, A. M. Edwards, I. Ruczinski, F. M. Poulsen, B. B. Kragelund, S. W. Michnick, F. Chiti, Y. Bai, S. J. Hagen, L. Serrano, M. Oliveberg, D. P. Raleigh, P. Wittung-Stafshede, S. E. Radford, S. E. Jackson, T. R. Sosnick, S. Marqusee, A. R. Davidson, K. W. Plaxco, Protein folding: Defining a "standard" set of experimental conditions and a preliminary kinetic data set of two-state proteins. *Protein Sci.* **14**, 602–616 (2005).

18. E. Braselmann, J. L. Chaney, P. L. Clark, Folding the proteome. *Trends Biochem. Sci.* **38**, 337–344 (2013).

19. L. Brocchieri, S. Karlin, Protein length in eukaryotic and prokaryotic proteomes. *Nucleic Acids Res.* **33**, 3390–3400 (2005).

20. D. Sarkar, P. Kang, S. O. Nielsen, Z. Qin, Non-arrhenius reaction-diffusion kinetics for protein inactivation over a large temperature range. *ACS Nano* **13**, 8669–8679 (2019).

21. Y. Feng, G. De Franceschi, A. Kahraman, M. Soste, A. Melnik, P. J. Boersema, P. P. de Laureto, Y. Nikolaev, A. P. Oliveira, P. Picotti, Global analysis of protein structural changes in complex proteomes. *Nat. Biotechnol.* **32**, 1036–1044 (2014).

22. P. To, B. Whitehead, H. E. Tarbox, S. D. Fried, Nonrefoldability is pervasive across the *E. coli* proteome. *J. Am. Chem. Soc.* **143**, 11435–11448 (2021).

23. S. Schopper, A. Kahraman, P. Leuenberger, Y. Feng, I. Piazza, O. Müller, P. J. Boersema, P. Picotti, Measuring protein structural changes on a proteome-wide scale using limited proteolysis-coupled mass spectrometry. *Nat. Protoc.* **12**, 2391–2410 (2017).

24. L. Malinovska, V. Cappelletti, D. Kohler, I. Piazza, T.-H. Tsai, M. Pepelnjak, P. Stalder, C. Dörig, F. Sesterhenn, F. Elsässer, L. Kralickova, N. Beaton, L. Reiter, N. de Souza, O. Vitek, P. Picotti, Proteome-wide structural changes measured with limited proteolysis-mass spectrometry: An advanced protocol for high-throughput applications. *Nat. Protoc.* **18**, 659–682 (2023).

25. F. J. O'Reilly, J. Rappsilber, Cross-linking mass spectrometry: Methods and applications in structural, molecular and systems biology. *Nat. Struct. Mol. Biol.* **25**, 1000–1008 (2018).

26. D. Pan, A. Brockmeyer, F. Mueller, A. Musacchio, T. Bange, Simplified protocol for cross-linking mass spectrometry using the MS-cleavable cross-linker DSBU with efficient cross-link identification. *Anal. Chem.* **90**, 10990–10999 (2018).

27. F. Herzog, A. Kahraman, D. Boehringer, R. Mak, A. Bracher, T. Walzthoeni, A. Leitner, M. Beck, F.-U. Hartl, N. Ban, L. Malmström, R. Aebersold, Structural probing of a protein phosphatase 2A network by chemical cross-linking and mass spectrometry. *Science* **337**, 1348–1352 (2012).

28. P. To, Y. Xia, S. O. Lee, T. Devlin, K. G. Fleming, S. D. Fried, A proteome-wide map of chaperone-assisted protein refolding in a cytosol-like milieu. *Proc. Natl. Acad. Sci. U.S.A.* **119**, e2210536119 (2022).

29. Y. Benjamini, Y. Hochberg, Controlling the false discovery rate: A practical and powerful approach to multiple testing. *J. R. Stat. Soc. Series B Stat. Methodol.* **57**, 289–300 (1995).

30. J. M. A. Bullock, J. Schwab, K. Thalassinos, M. Topf, The importance of non-accessible crosslinks and solvent accessible surface distance in modeling proteins with restraints from crosslinking mass spectrometry. *Mol. Cell. Proteomics* **15**, 2491–2500 (2016).

31. A. C. Tsolis, N. C. Papandreou, V. A. Iconomidou, S. J. Hamodrakas, A consensus method for the prediction of "Aggregation-Prone" peptides in globular proteins. *PLOS ONE* **8**, e54175 (2013).

32. J. I. Sułkowska, J. K. Noel, J. N. Onuchic, Energy landscape of knotted protein folding. *Proc. Natl. Acad. Sci. U.S.A.* **109**, 17783–17788 (2012).

33. L. Salicari, M. Baiesi, E. Orlandini, A. Trovato, Folding kinetics of an entangled protein. *PLOS Comput. Biol.* **19**, e1011107 (2023).

34. Z. Guo, D. Thirumalai, Kinetics of protein folding: Nucleation mechanism, time scales, and pathways. *Biopolymers* **36**, 83–102 (1995).

35. D. Thirumalai, S. A. Woodson, Kinetics of folding of proteins and RNA. *Acc. Chem. Res.* **29**, 433–439 (1996).

36. D. Thirumalai, D. K. Klimov, S. A. Woodson, Kinetic partitioning mechanism as a unifying theme in the folding of biomolecules. *Theor. Chem. Acta* **96**, 14–22 (1997).

37. W. L. Jorgensen, J. Chandrasekhar, J. D. Madura, R. W. Impey, M. L. Klein, Comparison of simple potential functions for simulating liquid water. *J. Chem. Phys.* **79**, 926–935 (1983).

38. A. E. Bryson, W. F. Denham, A steepest-ascent method for solving optimum programming problems. *J. Appl. Mech.* **29**, 247–257 (1962).

39. E. J. Haug, J. S. Arora, K. Matsui, A steepest-descent method for optimization of mechanical systems. *J. Optim. Theory Appl.* **19**, 401–424 (1976).

40. T. Darden, D. York, L. Pedersen, Particle mesh Ewald: An N·log(N) method for Ewald sums in large systems. *J. Chem. Phys.* **98**, 10089–10092 (1993).

41. S. Nosé, A unified formulation of the constant temperature molecular dynamics methods. *J. Chem. Phys.* **81**, 511–519 (1984).

42. W. G. Hoover, Canonical dynamics: Equilibrium phase-space distributions. *Phys. Rev. A. (Coll Park)* **31**, 1695–1697 (1985).

43. M. Parrinello, A. Rahman, Polymorphic transitions in single crystals: A new molecular dynamics method. *J. Appl. Phys.* **52**, 7182–7190 (1981).

44. B. Hess, H. Bekker, H. J. C. Berendsen, J. G. E. M. Fraaije, LINCS: A linear constraint solver for molecular simulations. *J. Comput. Chem.* **18**, 1463–1472 (1997).

45. M. J. Abraham, T. Murtola, R. Schulz, S. Páll, J. C. Smith, B. Hess, E. Lindah, Gromacs: High performance molecular simulations through multi-level parallelism from laptops to supercomputers. *SoftwareX* **1–2**, 19–25 (2015).

46. J. Huang, S. Rauscher, G. Nawrocki, T. Ran, M. Feig, B. L. De Groot, H. Grubmüller, A. D. MacKerell, CHARMM36m: An improved force field for folded and intrinsically disordered proteins. *Nat. Methods* **14**, 71–73 (2016).

47. W. Niemyska, K. C. Millett, J. I. Sulkowska, GLN: A method to reveal unique properties of lasso type topology in proteins. *Sci. Rep.* **10**, 15186 (2020).

48. M. B. B. Gutierres, C. B. C. Bonorino, M. M. Rigo, ChaperISM: Improved chaperone binding prediction using position-independent scoring matrices. *Bioinformatics* **36**, 735–741 (2020).

49. P. Eastman, J. Swails, J. D. Chodera, R. T. McGibbon, Y. Zhao, K. A. Beauchamp, L. P. Wang, A. C. Simmonett, M. P. Harrigan, C. D. Stern, R. P. Wiewiora, B. R. Brooks, V. S.

Pande, OpenMM 7: Rapid development of high performance algorithms for molecular dynamics. *PLOS Comput. Biol.* **13**, e1005659 (2017).

50. D. Arthur, S. Vassilvitskii, “K-means++: The advantages of careful seeding,” in *Proceedings of the Annual ACM-SIAM Symposium on Discrete Algorithms* (Society for Industrial and Applied Mathematics, 2007), pp. 1027–1035.

51. S. Röblitz, M. Weber, Fuzzy spectral clustering by PCCA+: Application to Markov state models and data classification. *Adv. Data Anal. Classif.* **7**, 147–179 (2013).

52. J.-H. Prinz, H. Wu, M. Sarich, B. Keller, M. Senne, M. Held, J. D. Chodera, C. Schütte, F. Noé, Markov models of molecular kinetics: Generation and validation. *J. Chem. Phys.* **134**, 174105 (2011).

53. M. K. Scherer, B. Trendelkamp-Schroer, F. Paul, G. Pérez-Hernández, M. Hoffmann, N. Plattner, C. Wehmeyer, J. H. Prinz, F. Noé, PyEMMA 2: A software package for estimation, validation, and analysis of markov models. *J. Chem. Theory Comput.* **11**, 5525–5542 (2015).

54. J. N. Onuchic, Z. Luthey-Schulten, P. G. Wolynes, THEORY OF PROTEIN FOLDING: The energy landscape perspective. *Annu. Rev. Phys. Chem.* **48**, 545–600 (1997).

55. Y. Jiang, Y. Xia, I. Sitarik, P. Sharma, H. Song, S. D. Fried, E. P. O’Brien, Protein misfolding involving entanglements providesa structural explanation for the origin of stretched-exponential refolding kinetics. *Sci. Adv.* **11**, eads7379 (2025).

56. S. Gosavi, L. L. Chavez, P. A. Jennings, J. N. Onuchic, Topological frustration and the folding of interleukin-1β. *J. Mol. Biol.* **357**, 986–996 (2006).

57. M. Y. Sherman, S. B. Qian, Less is more: Improving proteostasis by translation slow down. *Trends Biochem. Sci.* **38**, 585–591 (2013).

58. T. Kiefhaber, H. H. Kohler, F. X. Schmid, Kinetic coupling between protein folding and prolyl isomerization. I. Theoretical models. *J. Mol. Biol.* **224**, 217–229 (1992).

59. E. A. Kikis, "The intrinsic and extrinsic factors that contribute to proteostasis decline and pathological protein misfolding" in *Advances in Protein Chemistry and Structural Biology* (Elsevier Ltd., 2019), vol. 118.

60. P. Salahuddin, M. K. Siddiqi, S. Khan, A. S. Abdelhameed, R. H. Khan, Mechanisms of protein misfolding: Novel therapeutic approaches to protein-misfolding diseases. *J. Mol. Struct.* **1123**, 311–326 (2016).

61. A. Lafita, P. Tian, R. B. Best, A. Bateman, Tandem domain swapping: Determinants of multidomain protein misfolding. *Curr. Opin. Struct. Biol.* **58**, 97–104 (2019).

62. F. Yu, S. E. Haynes, G. C. Teo, D. M. Avtonomov, D. A. Polasky, A. I. Nesvizhskii, Fast quantitative analysis of timsTOF PASEF data with MSFragger and IonQuant. *Mol. Cell. Proteomics* **19**, 1575–1585 (2020).

63. E. Manriquez-Sandoval, J. Brewer, G. Lule, S. Lopez, S. D. Fried, FLiPPR: A processor for limited proteolysis (LiP) mass spectrometry data sets built on FragPipe. *J. Proteome Res.* **23**, 2332–2342 (2024).

64. M. L. Mendes, L. Fischer, Z. A. Chen, M. Barbon, F. J. O'Reilly, S. H. Giese, M. Bohlke-Schneider, A. Belsom, T. Dau, C. W. Combe, M. Graham, M. R. Eisele, W. Baumeister, C. Speck, J. Rappsilber, An integrated workflow for crosslinking mass spectrometry. *Mol. Syst. Biol.* **15**, e8994 (2019).
